# A New Approach to Developing Long-Acting Injectable Formulations of Anti-HIV Drugs: Poly(Ethylene Phosphoric Acid) Block Copolymers Increase the Efficiency of Tenofovir against HIV-1 in MT-4 Cells

**DOI:** 10.3390/ijms22010340

**Published:** 2020-12-30

**Authors:** Ilya Nifant’ev, Andrei Siniavin, Eduard Karamov, Maxim Kosarev, Sergey Kovalchuk, Ali Turgiev, Sergey Nametkin, Vladimir Bagrov, Alexander Tavtorkin, Pavel Ivchenko

**Affiliations:** 1 Chemistry Department, M.V. Lomonosov Moscow State University, 1–3 Leninskie Gory, 119991 Moscow, Russia; komrad.kosarev.maksim@gmail.com (M.K.); sn2711@gmail.com (S.N.); vlabag@yandex.ru (V.B.); phpasha1@yandex.ru (P.I.); 2 A.V. Topchiev Institute of Petrochemical Synthesis RAS, 29 Leninsky Pr., 119991 Moscow, Russia; tavtorkin@yandex.ru; 3 Faculty of Chemistry, National Research University Higher School of Economics, Miasnitskaya Str. 20, 101000 Moscow, Russia; 4 N.F. Gamaleya National Research Center for Epidemiology and Microbiology MHRF, 18 Gamaleya Str., 123098 Moscow, Russia; andreysi93@ya.ru (A.S.); karamov2004@yandex.ru (E.K.); turgiev@ld.ru (A.T.); 5 Shemyakin-Ovchinnikov Institute of Bioorganic Chemistry, Russian Academy of Sciences, 117997 Moscow, Russia; xerx222@gmail.com

**Keywords:** AIDS, cART, controlled release, HIV, injectable formulations, long-acting drugs, poly(phosphoric acid), polyphosphoesters, PrEP, ring-opening polymerization, tenofovir

## Abstract

Despite the world’s combined efforts, human immunodeficiency virus (HIV), the causative agent of AIDS, remains one of the world’s most serious public health challenges. High genetic variability of HIV complicates the development of anti-HIV vaccine, and there is an actual clinical need for increasing the efficiency of anti-HIV drugs in terms of targeted delivery and controlled release. Tenofovir (TFV), a nucleotide-analog reverse transcriptase inhibitor, has gained wide acceptance as a drug for pre-exposure prophylaxis or treatment of HIV infection. In our study, we explored the potential of tenofovir disoproxil (TFD) adducts with block copolymers of poly(ethylene glycol) monomethyl ether and poly(ethylene phosphoric acid) (mPEG-*b*-PEPA) as candidates for developing a long-acting/controlled-release formulation of TFV. Two types of mPEG-*b*-PEPA with numbers of ethylene phosphoric acid (EPA) fragments of 13 and 49 were synthesized by catalytic ring-opening polymerization, and used for preparing four types of adducts with TFD. Antiviral activity of [mPEG-*b*-PEPA]TFD or tenofovir disoproxil fumarate (TDF) was evaluated using the model of experimental HIV infection in vitro (MT-4/HIV-1_IIIB_). Judging by the values of the selectivity index (SI), TFD exhibited an up to 14-fold higher anti-HIV activity in the form of mPEG-*b*-PEPA adducts, thus demonstrating significant promise for further development of long-acting/controlled-release injectable TFV formulations.

## 1. Introduction

Human immunodeficiency virus (HIV) is an infectious agent of acquired immunodeficiency syndrome (AIDS). The number of HIV-infected persons grows every year, from few cases in 1981 to ~38 million in 2019; a total of ~33 million people have died of the virus to date [1]. The development of anti-HIV vaccines has not yet led to success, therefore, antiviral therapy remains a major means of combating HIV. Combination antiretroviral therapy (cART; three drugs at a time, to be administered once daily) is a universally recognized means of the first-line treatment for HIV infection, making it possible to achieve suppression of viral replication and restoration of CD4 cell counts in the majority of patients. The same strategy was adopted for pre-exposure prophylaxis (PrEP), consisting of a two-drug regimen in once-daily oral dosing [2]. Although HIV-infection is currently manageable and no longer viewed as a fatal disease, this obvious progress has its reverse side, since fewer than 62% of patients maintain the ≥90% adherence which is required for optimal viral suppression. PrEP, recommended by the World Health Organization (WHO) for high-risk populations with HIV incidence of ≥3%, may have adherence of as low as 28%. Not only does compromised adherence hamper the therapeutic and prophylactic outcomes of cART and PrEP, it also becomes a major factor contributing to HIV-1 drug resistance [3]. Clearly, ending HIV/AIDS (a goal set by the United Nations for 2030) will not be possible unless and until the problem of suboptimal adherence is solved, thereby improving success rates of cART/PrEP and eliminating the negative impact of emergence of resistant strains on public health.

As an alternative to the more conventional oral regimen, long-acting injectables can be administered on a more infrequent basis, reducing patient non-compliance and minimizing the development of drug resistance. As of today, only a few long-acting injectable formulations of antiretroviral drugs have been approved by regulatory agencies or are currently under preclinical/clinical evaluation [2,3,4,5,6]. However, the majority of the investigated antiretroviral agents are not well suited for formulation as long-acting injectables due to suboptimal physicochemical properties limiting their formulation as conventional drug suspensions, as well as insufficient antiviral potency resulting in high monthly dosing requirements. The identification of single vehicles capable of dissolving multiple drugs for solution-based long-acting injections provides a number of hurdles, especially when attempting to overlay various partition coefficients to maintain the dose ratios over extended periods [7,8,9]. It is thus clear that novel formulation approaches capable of achieving extended-duration pharmacokinetics for molecules of diverse physicochemical properties, at practical injection volumes and with a limited number of injections, are highly desirable.

**Scheme 1:**
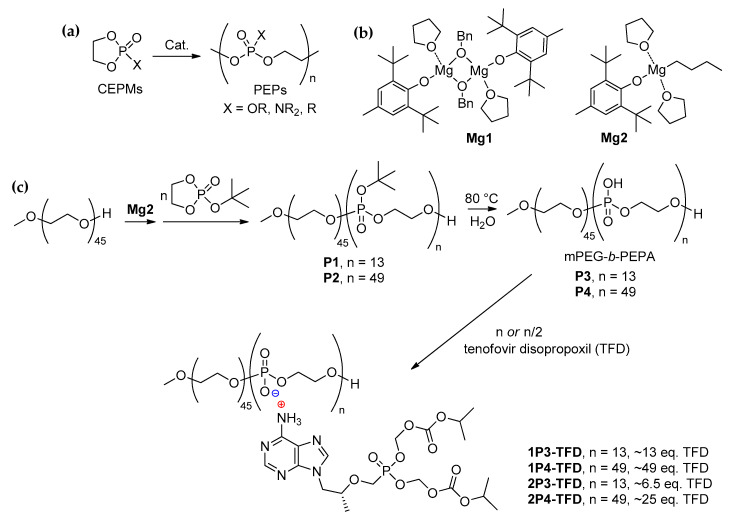
(**a**) Ring-opening polymerization (ROP) of cyclic ethylene phosphate monomers (CEPMs); (**b**) 2,6-di-*tert*-butyl-4-methylphenoxy (BHT)-Mg complexes, efficient catalysts of living ROP of CEPMs; (**c**) Controlled synthesis of poly(ethylene phosphoric acid) (PEPA) block copolymers and adducts with tenofovir disoproxil (TFD) [29].

An intriguing opportunity would lie within the development of long-acting, synthetic scaffolds for delivering antiretrovirals. Those scaffolds should be designed with the goal to reduce the frequency of dosing and, furthermore, hold the possibility of potential targeting of the drugs to key HIV residual sites [10]. The fragments of poly(ethylene glycol) (PEG)—a hydrophilic polymer improving solubility and acting as a transporter—are widely used in development of such scaffolds. A promising alternative to PEG is comprised by poly(ethylene phosphates) (PEPs), which are structurally similar to the phosphoester backbone of polynucleic acids: chemically versatile, biocompatible, and biodegradable; those properties allow the development of tailored materials suitable for different uses, including as polymers for biomedical applications [11,12,13,14,15,16,17,18,19,20,21,22,23,24,25,26,27]. PEP is usually prepared by the catalytic ring-opening polymerization (ROP) of cyclic ethylene phosphate monomers (CEPMs) (Scheme 1a). Given that catalytic ROP can be carried out in living mode, various block copolymers based on PEPs can be obtained. Recently, we found that efficient catalysts of this process are heteroleptic Mg complexes of 2,6-di-*tert*-butyl-4-methylphenol (BHT-H) **Mg1** and **Mg2** [17,28,29,30] (Scheme 1b).

Copolymers of poly(ethylene phosphoric acid)s (PEPA) that can be obtained by different methods [31,32,33,34,35,36,37,38], are polyacids potentially able to bind and deliver organic bases, including drugs. Despite the obvious promise of PEPA for different biomedical applications [27,36,37,39,40,41,42,43,44], the ability of PEPA-containing polymers to act as a carrier of antiviral drugs with basic functional groups have not been studied to date.

In developing approaches to the use of PEPA copolymers for delivery of basic antiviral drugs, precision control on the number of phosphate fragments must be implemented. In our recent work, we proposed an efficient method of the synthesis of PEPA-containing (co)polymers using living block copolymerization of the sterically hindered *tert*-butyl ethylene phosphate (^t^BuOEP), catalyzed by heteroleptic magnesium 2,6-di-*tert*-butyl-4-methylphenol (BHT) complexes **Mg1** and **Mg2** (Scheme 1b), with subsequent thermolysis of ^t^BuOEP (co)polymers in aqueous media at 80 °C [29] (Scheme 1c).

Tenofovir (TFV) is an antiviral drug approved for treating HIV and Hepatitis B. TFV is administered orally as the prodrugs tenofovir disoproxil fumarate (TDF) or tenofovir alafenamide (TAF) which then are hydrolized to TFV. TDF has been part of first-line anti-HIV therapy since 2001; it has also been approved for PrEP (as monotherapy or co-formulated with emtricitabine) [45]. In this communication, we report that newly obtained hydrophilic block-copolymers of methylated poly(ethylene glycol) (mPEG) and PEPA give rise of the antiviral activity of TFV against HIV-1_IIIB_ in MT-4 cells.

## 2. Results

### 2.1. Preparation of mPEG-b-PEPA Copolymers and Copolymer Complexes with Tenofovir Disopropoxil

**Figure 1:**
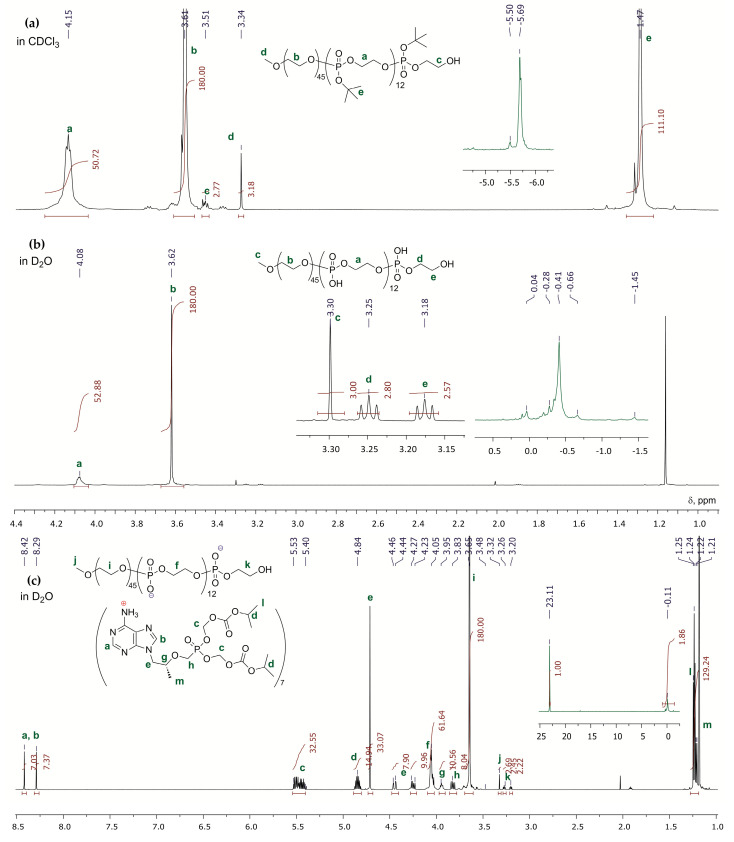
^1^H (black) and ^31^P (green) NMR spectra of **P1** (**a**), **P3** (**b**) and **2P3-TFD** (**c**).

To form the active initiator of the ROP, heteroleptic complex **Mg2** (Scheme 1b) was treated by one equivalent of mPEG-2000 (~45 –CH_2_CH_2_O– units) in CH_2_Cl_2_. Then, 15 or 60 equivalents of *^t^*BuOEP were added. After reaching 80% conversion of *^t^*BuOEP (control by ^1^H and ^31^P NMR spectroscopy), polymerization was terminated by the addition of AcOH. Copolymers of the formula MeO(CH_2_CH_2_O)_45_(*^t^*BuOEP)_n_H (**P1** and **P2**, n = 13 and 49, respectively, Scheme 1c) were separated and characterized (see Figure 1 and Section S2 in the Supplementary Materials). Then, copolymers **P1** and **P2** were hydrolyzed at 80 °C within 45 min with a formation of PEPA copolymers **P3** and **P4** having the same comonomer ratios. Polyphosphate fragments in these copolymers were partially branched (see Figure 1 and Section S2 in the Supplementary Materials). Concentrations of phosphate groups in aqueous solutions of **P3** and **P4** were determined using ^31^P NMR spectroscopy with the addition of trimethyl phosphate (TMP) as an internal standard (for details, see Section S2 in the Supplementary Materials).

Thus, obtained aqueous **P3** and **P4** solutions (concentration of phosphate groups 103 and 93 μmol/g, respectively) were used for the preparation of adducts with TFD (free base, obtained by the reaction of commercial drug TDF with NaOH, see Section S2 in the Supplementary Materials). Concentrations and ratios of ethylene phosphate groups of copolymers and TFD were determined using ^31^P NMR spectroscopy with the addition of TMP as an internal standard (for details, see Section 4.1 and Section S2 in the Supplementary Materials). As a result, aqueous solutions of four complexes with TFD concentration of 0.05 mol/g were obtained, namely: **1P3-TFD**, **1P4-TFD** (1:1 ethylene phosphate/TFD molar ratio), **2P3-TFD**, and **2P4-TFD** (2:1 ethylene phosphate/TFD molar ratio). ^1^H NMR spectra of **2P3-TFD** are presented in Figure 1c (NMR assignment for TFD is based on the data reported previously [46]).

### 2.2. Cytotoxicity of mPEG-b-PEPA Adducts of TFD

**Figure 2:**
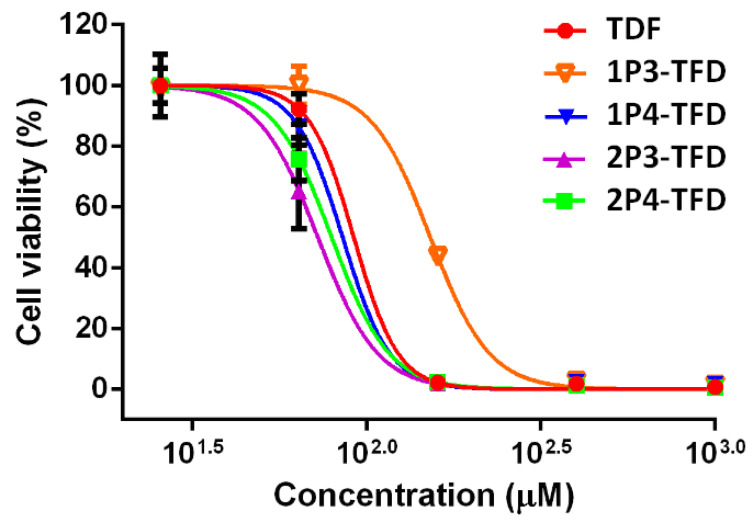
Cytotoxicity of TDF and TFD adducts. MT-4 cells were incubated with the compounds for 72 h, after which viability was determined using the MTT test. For TDF, absorbance values corresponding to 100% viability were obtained by culturing untreated cells; in the case of the adducts, the cells were cultured in the presence of equivalent concentrations of the corresponding mPEG-*b*-PEPA copolymers.

Cytotoxicity was assessed by the MTT test in MT-4 cells exposed for 72 h to TDF or TFD adducts differing in PEPA chain length and copolymer to TFD ratio. All compounds exhibited comparable cytotoxicity, with CC_50_ values in the range 72.5–152.4 µM (Table 1 and Figure 2). Notably, 1:1 adduct **1P3-TFD** was twice less toxic than other compounds, exhibiting an IC_50_ of 152.4 µM (a concentration lethal to the cells for all other compounds), about 40% cells retaining viability. No cytotoxic effects were observed in MT-4 cells incubated with equivalent amounts of the corresponding block copolymers **P3** and **P4**.

### 2.3. The Ability of TDF or TFD Adducts to Induce Apoptosis

**Figure 3:**
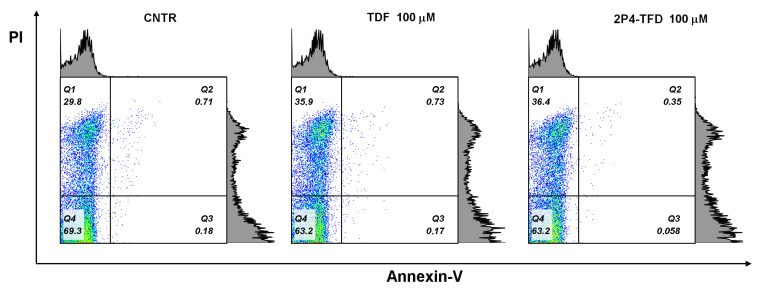
Study of compound-induced apoptosis and necrosis. Peripheral blood mononuclear cells (PBMC) were treated with the compounds (100 µM) for 24 h. Then, the cells were stained with the mixed anti-CD3/Annexin-V/PI and analyzed by flow cytometry. Representative profiles of apoptosis and necrosis are shown for the CD3^+^ cell population. Quadrants: living cells (Q4, Annexin V−/PI−), early apoptosis (Q3, Annexin V+/PI−), late apoptosis (Q2, Annexin V+/PI+), and necrosis (Q1, Annexin V−/PI +).

To check whether the difference in CC_50_ values was due to variations in apoptogenic potential of the compounds, we performed a flow cytometry study in peripheral blood mononuclear cells (PBMC). Lymphocytes, in which apoptosis and necrosis were assessed, were identified by CD3 expression. Apoptotic cells were quantified by flow cytometry after staining of phosphatidylserines translocated to the outer leaflet of the membrane with Annexin V. In order to discriminate apoptotic cells from necrotic and intact cells, Annexin V was combined with propidium iodide (PI) that stains permeabilized cells. Unlabeled cells were considered viable, whereas Annexin V-positive and Annexin V/PI-positive cells, respectively, were early apoptotic and late apoptotic. Figure 3 demonstrates that there were no significant differences between TDF and the adducts (each taken at 100 µM) in the ability to induce apoptosis in CD3^+^ cells throughout 24 h of incubation.

### 2.4. Antiviral Activity of mPEG-b-PEPA Adducts of TFD

**Figure 4:**
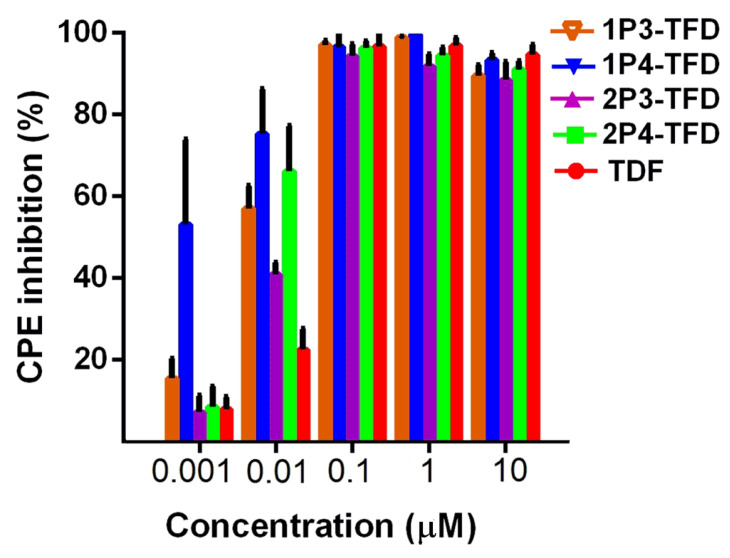
Suppression of HIV_IIIB_-induced cytopathic effects (CPE) by TDF or TFD adducts. MT-4 cells were treated at 37 °C with variable concentrations of the compounds (or their vehicle) for 1 h prior to inoculation with the virus; CPE was measured after 5 days of cell culturing. The levels of CPE observed in infected non-treated cells were taken as 0% of CPE inhibition; values obtained in compound-treated wells were expressed as percentages of those measured for uninfected cells. Equivalent concentrations of the corresponding diblock copolymers did not produce any CPE suppression (data not shown). The results obtained using as a readout the level of HIV-1 p24 production were closely similar (data not shown).

Antiviral activity was studied in the lymphoblastoid T-cell line MT-4 infected with the reference strain HIV-1_IIIB_. Compound concentrations required for 50% virus suppression (IC_50_) were determined in a 5-day assay based on the virus-induced cytopathic effects (CPE). TDF suppressed HIV-1_IIIB_ replication with an IC_50_ of 19 nM, whereas all TFD adducts were more active, with IC_50_ values in the range 1.3–11 nM (Table 2 and Figure 4). The highest activity was exhibited by the adduct **2P3-TFD**.

The selectivity index (SI) is a ratio that measures the window between cytotoxicity and antiviral activity, obtained by dividing CC_50_ by IC_50_. The higher SI, the more efficient and safe a drug would be during in vivo treatment for a given viral infection [47]. As Table 3 shows, SI value of mPEG-*b*-PEPA_49_ (1:1) exceeds that of TDF considerably (~14×), suggesting that this adduct shows significant promise for further development as a pharmaceutical.

### 2.5. Stability of mPEG-b-PEPA Adducts of TFD in Human Serum

**Figure 5:**
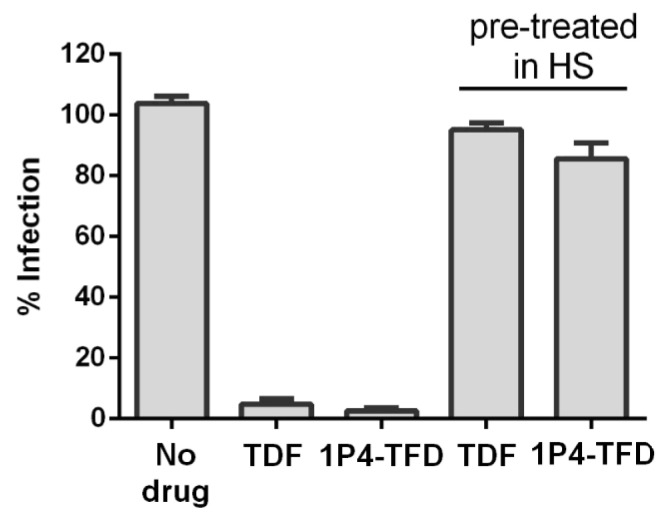
Comparative evaluation of the susceptibility of TDF and **1P4-TFD** adduct to human serum-induced inactivation. The efficacy of the compounds against HIV-1_IIIB_ infection was assessed in MT-4 cells at 10× IC_50_ following pretreatment with human serum for 30 min.

TDF was shown previously to lose its antiviral potency on in vitro exposure to human plasma and serum [48]. We therefore decided to assess whether TFD adducts are as susceptible to biodegradation as TDF. For this reason, in vitro anti-HIV activity of the compounds was assessed in MT-4 cells using a 5-day infectivity assay, with pretreatment in the growth medium (standard conditions) or 100% human serum (HS) to mimic plasma exposure. As shown in Figure 5, TDF and **1P4-TFD** adduct were equally susceptible to HS-induced degradation, whereas, under standard conditions, the antiviral activity was retained. Thus, substituting mPEG-*b*-PEPA for fumaric acid failed to protect TFD from cleavage by esterases present in human plasma and serum.

### 2.6. Metabolic Conversions of TDF and mPEG-b-PEPA Adducts of TFD in MT-4 Cells: A Preliminary Study

**Figure 6:**
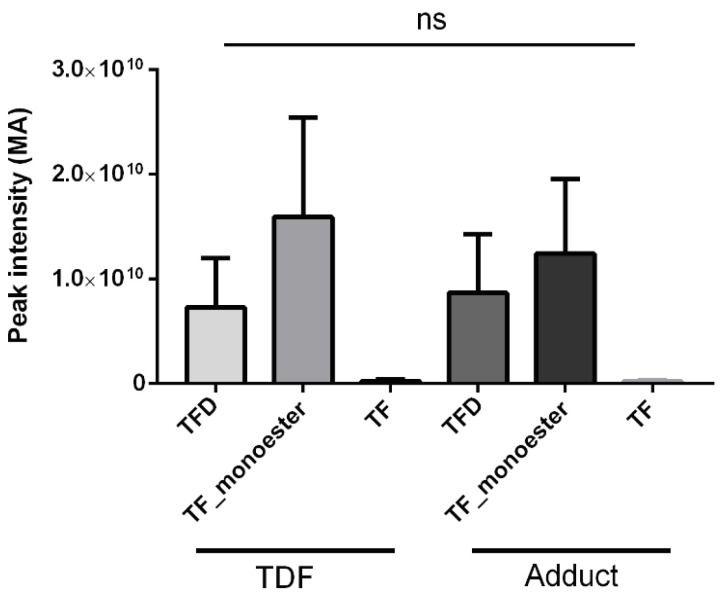
**TDF-** and **1P4-TFD**-derived metabolites formed in MT-4 cells after 4 h of incubation. Cell extracts were prepared following incubation with 1 µM each of TDF or **1P4-TFD** (adduct) and intracellular metabolites, determined by LC-MS/MS. Shown are the levels of TFD, TF_monoester, and tenofovir base (TFV). Data for the graphs are means of three independent experiments (six runs/measurements per experiment) ± SD. No significant (ns) differences between the compounds could be detected in either the spectrum or amount of the metabolites (*p* = 3; one-way ANOVA and Tukey’s post hoc test).

Initiation of HIV reverse transcription (defined as reaching 1% of its total progression) occurs as early as 3 h after the virus-to-cell-membrane fusion [49] that is preceded by HIV attachment to its cellular receptors; fusion events are detected after a time lag of 10–15 min following virus and cell co-culture [50], reaching maximum with a t_1/2_ of 19 min [51] (see also [52]). We therefore examined transformations of TDF and **1P4-TFD** by determining their metabolites in cell extracts following 4 h of incubation with MT-4 cells. In both cases, the most abundant metabolite was the monoester tenofovir isopropoxyl, but the differences between the two compounds in the metabolic profiles were insignificant (Figure 6).

## 3. Discussion

TFV [(R)-9-(2-phosphonomethoxypropyl)adenine] contains a phosphonate group that is equivalent to the first phosphate group present in natural monophosphate nucleotides. Intracellular phosphorylation results in the formation of tenofovir diphosphate (TDP), which is incorporated into viral DNA by HIV reverse transcriptase and acts as a DNA chain terminator. The negative charges of the phosphonate moiety of TFV (reducing its ability to permeate cells and limiting absorption/oral bioavailability) are eliminated by introduction of two isopropyloxymethylcarbonyl groups [53]. The resulting molecule, a TDP pre-prodrug, is marketed in the form of a fumaric acid salt known as TDF; its oral administration allows systemic delivery of TFV [54]. Another such pre-prodrug, an isopropylalaninyl phenyl ester of TFV (TAF), which is significantly more stable in blood and plasma, was designed subsequently [55]. Both TDP pre-prodrugs are widely used for the treatment and prevention of HIV infection, either alone or in combination with other antiretroviral agents [48].

Developing long-acting injectable formulations that achieve extended-duration pharmacokinetics for antiretrovirals is a currently topical problem in the fight against HIV/AIDS [2,56]. In the case of TFV, such formulations may be obtained by preparing salts of a TFV base with a polymeric acid; their intramuscular administration would then form tissue depots, gradually releasing the base and making it available for internalization by cells.

We have recently developed an approach to synthesis of PEPAs and PEPA-based block copolymers [29] and demonstrated their biocompatibility [41]. Hypothetically, salts formed by by TFV base with such copolymers offer an opportunity to regulate the rate of TFV release by varying the length of PEPA chains and acid to base molar ratios.

This work is the first report of our experiments aimed at probing the validity of this hypothesis. We synthesized two mPEG-*b*-PEPA copolymers differing in the PEPA chain length and prepared their adducts with TFD; the effect of such modifications on in vitro anti-HIV activity was further assessed in MT-4 cells infected with HIV-1_IIIB_. With each polymer, two types of adducts were prepared, differing in phosphate to TFD molar ratio (2:1 and 1:1).

As shown in Table 1, the rank order of potency of the compounds under study in suppressing HIV-1 replication was as follows: **1P4-TFD** > **2P4-TFD** > **1P3-TFD** > **2P3-TFD** > TDF. The antiviral potency seemed to be directly proportional to the length of the PEPA chain and the extent of its ‘saturation’ with TFD.

Since TDP is responsible for both the anti-HIV potency and cytotoxicity of TDF and TAF [53], we expected to obtain the same rank order for the cytotoxicity of the compounds under study. As follows from Table 2, however, the two rank orders did not coincide: **2P3-TFD** > **2P4-TFD** > **1P4-TFD** > TDF> **1P3-TFD**. Consequently, yet another rank order was obtained for SI values: **1P4-TFD** > **1P3-TFD** > **2P4-TFD** > **2P3-TFD** > TDF. A flow cytometry study of the apoptogenic potential revealed no differences between the compounds (Figure 3).

Given the fact that none of the respective mPEG-*b*-PEPAs exhibited either cytotoxicity or anti-HIV activity (data not shown), it would seem that the observed discrepancies are related to the heterogeneity of the dispersions formed by the adducts in aqueous medium. Indeed, TFD is hydrophobic [57] and its association with PEPA is likely to confer amphipathicity to the adducts, which is prerequisite to micelle formation [58]. Micelles are dynamic structures that readily disassemble and change size on interaction with the surroundings, such as the extracellular matrix [59,60]. Under certain conditions, they may form single amphipathic units acting on cell membranes as surfactants and exerting toxic effects [59].

The assumption that the adducts under study are micellar in nature is not contradicted by our finding that the anti-HIV activity of the adducts correlated with the length of PEPA chains and their hydrophobicity (i.e., extent of saturation with TFD). Similar observations were reported in the literature for poly(D,L-lactide-co-glycolide)-based micelles delivering bacitracin A [61] and gene complexes for transfection [62]. The instability of the dispersions formed by the adducts may also explain the unusually pronounced data scatter observed in our metabolic experiments, due to which we found no significant differences between TDF and **1P4-TFD** (Figure 6).

## 4. Materials and Methods

### 4.1. Synthesis of mPEG-b-PEPA and TFD Adducts

#### 4.1.1. General Experimental Remarks

All the synthetic and polymerization experiments were carried out under an argon atmosphere. Tetrahydrofuran (THF), diethyl ether, and triethylamine (Merck, Darmstadt, Germany) were refluxed with Na/benzophenone and distilled prior to use. Benzene (Merck, Darmstadt, Germany) was distilled with Na/benzophenone/dibenzo-18-crown-6. Pentane was refluxed for 10 h over sodium, distilled, and stored under an argon atmosphere over sodium wire. 2,6-Di-*tert*-butyl-4-methylphenol (BHT-H, ≥99%, Merck, Darmstadt, Germany), di-*n*-butylmagnesium (1.0 M solution in heptane, Merck, Darmstadt, Germany), mPEG-2000 (Merck, Darmstadt, Germany,) and acetic acid (≥99.9%, Acros Organics, Geel, Belgium) were used as purchased. *tert*-Butyl alcohol (99%, Merck, Darmstadt, Germany) was distilled over BaO and stored under argon. 2-Chloro-2-oxo-1,3,2-dioxaphospholane [63], 2-*tert*-butoxy-2-oxo-1,3,2-dioxaphospholane *^t^*BuOEP [17], and BHT-Mg precatalyst (BHT)Mg(*n*-Bu)(THF)_2_ (**Mg2**) [64] were prepared according to previously described methods (for details and NMR spectra, See Section S1 in the Supplementary Materials).

Size exclusion chromatography (SEC) was performed on an Agilent PL-GPC 220 chromatograph (Agilent Technologies, Santa Clara, CA, USA) equipped with a PLgel column, using DMF as an eluent (1 mL/min). The measurements were recorded with universal calibration according to poly(ethylene glycol) standards at 40 °C.

#### 4.1.2. Synthesis of mPEG-b-poly(^t^BuOEP)

Polymer **P1**. **Mg2** (100 mg, 0.225 mmol) was added to the solution of mPEG (M = 2000, 448 mg, 0.224 mmol) in CH_2_Cl_2_ (1.5 mL). After 3 h of stirring at 20 °C, *^t^*BuOEP (625 mg, 3.62 mmol) was added. After 30 h AcOH (20 mg) was added, the mixture was poured into pentane (10 mL). The polymer deposited was dissolved in CH_2_Cl_2_ (1 mL), re-precipitated again, separated, and dried at 50 °C (0.01 Torr) to constant weight. The yield was 894 mg (83%), pale-yellow sticky solid, *M_n_* (SEC) = 4.23·10^3^, *M*_n_ (NMR) = 3.61·10^3^, *Ð_M_* = 1.45. For ^1^H and ^31^P NMR spectra of **P1**, see Figures S8 and S9 in the Supplementary Materials.

Polymer **P2**. **Mg2** (111 mg, 0.25 mmol) was added to the solution of mPEG (M = 2000, 501 mg, 0.25 mmol) in CH_2_Cl_2_ (1.5 mL). After 3 h of stirring at 20 °C, *^t^*BuOEP (2.25 g, 12.49 mmol) was added. After 30 h AcOH (20 mg) was added, the mixture was poured into pentane (10 mL). The polymer deposited was dissolved in CH_2_Cl_2_ (1 mL), re-precipitated again, separated, and dried at 50 °C (0.01 Torr) to constant weight. The yield was 2.188 g (81%), pale-yellow sticky solid, *M_n_* (SEC) = 9.00·10^3^, *M*_n_ (NMR) = 8.08·10^3^, *Ð_M_* = 1.48. For ^1^H and ^31^P NMR spectra of **P2**, see Figures S10 and S11 in the Supplementary Materials.

#### 4.1.3. Preparation of mPEG-*b*-PEPA Solutions and TFD Adducts

Polymers **P3** and **P4**, common method. Suspension of **P1** or **P2** (316 mg or 205 mg, respectively) in H_2_O (10 mL) was heated for 45 min at 80 °C. The resulting opaque solution was washed by CH_2_Cl_2_ (4 × 1 mL), heated 2 h at 60 °C to eliminate organic solvents, cooled, degassed at 10 Torr, and filtered through PTFE membrane filter (0.45 µm). For ^1^H and ^31^P NMR spectra of **P3** and **P4**, see Figures S12–S15 in the Supplementary Materials. The molar concentrations of phosphate groups in polymer solutions were determined by ^31^P NMR spectroscopy using trimethyl phosphate (TMP) as an internal standard (for example, see Figure S16 in the Supplementary Materials).

TFD was prepared by neutralization of TDF (Drug Technology company, Khimki, Russian Federation) by aqueous NaHCO_3_ followed by the extraction with CH_2_Cl_2_ and evaporation of the organic phase (for ^1^H NMR spectrum see Figure S17 in the Supplementary Materials). Aqueous solutions of the polymer complexes of TFD (free base) with [TFD] = 0.05 mol/l were prepared by the addition of TFD sample weights to polymer solutions with subsequent dilution (if appropriate):

- **1P3-TFD**: 275 mg TFD, 1.220 g of **P3** solution; 1.395 g of the solution obtained, dilution by 1.605 g H_2_O.
- **1P4-TFD**: 225 mg TFD, 1.5 g of **P4** solution; 1.755 g of the solution obtained, dilution by 1.245 g H_2_O.
- **2P3-TFD**: 78 mg TFD, 2.92 g of **P3** solution.
- **2P4-TFD**: 78 mg TFD, 2.92 g of **P4** solution.

To determine molar concentrations of **P3**, **P4**, and TDF, TMP was added in 10:1 [TMP]/[OCH_2_CH_2_OP(O)O] ratio. For ^1^H and ^31^P NMR spectra of the adducts **1P3-TFD**–**2P4-TFD**, see Figures S18–S21 in the Supplementary Materials.

### 4.2. Cells and Viruses

#### 4.2.1. Cells

MT-4 cells (human HTLV-1 transformed T-lymphoblasts; NIH AIDS Reagent Program) were cultured in complete RPMI 1640 medium (Thermo Fisher Scientific, Waltham, MS, USA) supplemented with 10% fetal calf serum (FCS; Merck, Darmstadt, Germany), 1% GlutaMAX, and 1% penicillin-streptomycin (both from Thermo Fisher Scientific, Waltham, MS, USA). Peripheral blood mononuclear cells (PBMC) were isolated from the blood of HIV-seronegative donors by Ficoll^®^ Paque Plus (GE Healthcare, Chicago, IL, USA) gradient centrifugation. PBMC were cultured in complete RPMI 1640 medium containing 100 U/mL human recombinant interleukin-2 (Hoffmann–La Roche, Basel, Switzerland).

#### 4.2.2. Virus and Virus Titration

The viral stock of the reference strain HIV-1_IIIB_ (NIH AIDS Reagent Program) was obtained during acute infection of MT-4 cells. The virus was stored in aliquots at −80 °C.

The titer of the viral stock was determined as described in [65]. In brief, HIV-induced CPE were observed microscopically on day 5 days, and 50% cell culture infectious dose (CCID_50_) was obtained by end-point dilution of the virus in suspensions of MT-4 cells.

### 4.3. Cytotoxicity Assay

Cell viability was assessed by the MTT assay based on the ability of live cells to convert the water-soluble yellow 3-(4,5-dimethylthiazol-2-yl)-2,5-diphenyltetrazolium bromide (MTT) into insoluble purple intracellular crystals of MTT-formazan. The conversion efficiency is indicative of the general level of dehydrogenase activity of the cells under study, which is to a certain extent directly proportional to the concentration of viable cells [65]. MT-4 cells were mixed in 96-well microtiter plates (Corning, Corning, NY, USA) with various concentrations of test compounds in a final volume of 200 µl. After 72 h of incubation at 37 °C, 20 µL MTT (Merck, Darmstadt, Germany; 5 mg/mL) was added to each well of the plate and the plates were incubated at 37 °C for additional 4 h. The supernatants were removed, and 150 µl DMSO was added to each well. Absorbance was measured at 570 nm on a Hidex Sense microplate reader (Hidex, Turku, Finland) and the reference wavelength was 700 nm. Cell viability was expressed as a percentage of the signal from untreated cells (TDP) or mPEG-*b*-PEPA-treated cells (adducts). The concentration of compounds that reduced cell viability by 50% (CC_50_) was determined using non-linear regression analysis (Prism 6; GraphPad Software, San Diego, CA, USA).

### 4.4. Antiviral Activity Assay

Antiviral activity of the compounds under study was assessed in MT-4 by the inhibition of HIV-induced CPE and p24 production. HIV-induced CPE was measured colorimetrically, using the MTT test [65]. The viral core antigen p24 was measured by ELISA, using certified commercial test systems purchased from Vector-Best (Vector-Best, Novosibirsk, Russia), according to the manufacturer’s instructions. In brief, MT-4 cells (6 × 10^5^ cells/mL) were preincubated with the solutions of TDF or TFD adducts (concentrations of tenofovir in the range of 0.001–10.0 µM) in 96-well microtiter plates (Corning, Corning, NY, USA) for 1 h at 37 °C, after which they were inoculated with HIV-1_IIIB_ at 100 CCID_50_; CPE and p24 were measured after 5 days. Half-maximal inhibitory concentrations (IC_50_) of the compounds were derived from concentration-response curves, using non-linear regression analysis (Prism 6; GraphPad Software, San Diego, CA, USA). Selectivity indices (SI) were calculated using the formula SI = CC_50_/IC_50._

### 4.5. Apoptosis Assay

The ability of the compounds under study to induce cell apoptosis was studied in PMBC on a BD Biosciences FACS Calibur flow cytometer (BD Biosciences, San Jose, CA, USA) using APC Annexin V apoptosis detection kit with propidium iodide (Biolegend, San Diego, CA, USA) according to the manufacturer’s instructions. To determine the population of CD3^+^ lymphocytes, PMBC were stained with Alexa Fluor 488 anti-human CD3 antibody (Clone HIT3a; Sony Biotechnology, San Jose, CA, USA). PBMCs were incubated with the compounds under study for 24 h prior to apoptosis/necrosis assessment. Data were analyzed using FlowJo 10 software (BD Biosciences, San Jose, CA, USA).

### 4.6. Serum Treatment

Experiments assessing the antiviral activity were performed in MT-4 cells as described above in the presence of TDF or adducts TD, pretreated or mock treated for 30 min in AB human serum (HS; Thermo Fisher Scientific, Waltham, MS, USA). The HS was pooled from multiple donors; its final concentration was adjusted to 5% for all samples prior to infection [48].

### 4.7. Metabolism of TDF and mPEG-b-PEPA Adducts of TD in MT-4 Cells

Transformations of the compounds under study were assessed by identifying the parent drugs and their metabolites, using liquid chromatography tandem mass spectrometry (LC-MS/MS). MT-4 cells (10^6^ per 1 mL complete RPMI-1640 medium) were incubated with TDF or its adducts (1 µM each) for 4 h. At the end of the incubation, the cells were washed thrice with 1 mL of ice cold 1 mM phosphate-buffered saline (PBS), and metabolites were extracted overnight in 70% (*v*/*v*) methanol at −80 °C. Methanol extracts were dried at 37 °C using a miVac Quattro centrifuging evaporator (Genevac, UK) and resuspended in PBS. The extracts thus obtained were analyzed by the presence and intensity of peaks with *m*/*z* values characteristic of TFD and metabolites thereof [55,66,67]. LC-MS analysis was done on an Ultimate3000 RSLC nano LC system connected to QExactive Plus mass spectrometer (Thermo Fischer Scientific, Waltham, MS, USA). Dried cell extracts were dissolved, injected into a 20 × 0.1 mm PepMap C18 5 μm trap column (Thermo Fisher Scientific, Waltham, MS, USA) in the loading buffer (2% acetonitrile (ACN), 98% H_2_O, 0.1% trifluoroacetic acid (TFA)) at 10 μL/min flow, and separated at RT in a home-packed 300 × 0.1 mm fused-silica pulled emitter column packed with Reprosil PUR C18AQ 1.9 (Dr. Maisch, Ammerbuch-Entringen, Germany) [68]. Samples were eluted with a linear gradient of 80% ACN, 19.9% H_2_O, 0.1% formic acid (FA) (buffer B) in 99.9% H_2_O, 0.1% FA (solvent A) from 8 to 80% of solvent B in 15 min at 0.5 μL/min flow. MS1 spectra were recorded in positive mode with 70K resolution, 150–1500 scan range, 3e6 automatic gain control (AGC) target value, 50 ms maxIT. Peak areas were analyzed in Skyline (MacCoss Lab, Seattle, WA, USA) [69]. TFD, tenofovir monoester, and tenofovir base were detected, respectively, with m/z values of 520.1903, 404.1330, and 288.0856. Peak assignment was done using TFD and tenofovir standards (injected separately); tenofovir monoester was detected based on the parent ion isotope distribution conformity of that expected from the chemical composition as estimated by SkyLine. Statistical significance of the results was determined by one-way ANOVA test with a Tukey’s post hoc test for multiple comparisons.

## 5. Conclusions

We prepared mPEG-*b*-PEPA adducts with TFD, differing in the length of PEPA chains and the extent of their saturation with TFD. We found that all adducts were more active than TDF against HIV-1_IIIB_ in MT-4 cells; TFD 1:1 adduct with mPEG-*b*-PEPA_49_ exhibited a 14 times higher selectivity index. Thus, polyether–PEPA and polyester–PEPA block copolymers may well serve as a scaffolds for next-generation long-acting injectable formulations of antiretrovirals.

## Figures and Tables

**Table 1:** Cytotoxicity of tenofovir disoproxil fumarate (TDF) and poly(ethylene glycol) monomethyl ether and poly(ethylene phosphoric acid) (mPEG-*b*-PEPA) adducts of tenofovir disoproxil (TFD).

Compound	CC50 (μM)	95% CI	R2
**TDF**	91.9	71.2–118.6	0.98
**1P3-TFD**	152.4	143.9–161.4	0.99
**1P4-TFD**	85.3	73.4–99.2	0.99
**2P3-TFD**	72.5	61.1–85.9	0.96
**2P4-TFD**	79.4	71.1–88.7	0.99

**Table 2:** Antiviral activity of TDF and mPEG-b-PEPA adducts of TFD ^1^.

Compound	IC_50_ (μM)	95% CI
**TDF**	0.019	0.011–0.03
**1P3-TFD**	0.007	0.0053–0.0087
**1P4-TFD**	0.0013	0.0006–0.0028
**2P3-TFD**	0.011	0.01–0.017
**2P4-TFD**	0.006	0.004–0.008

^1^ Data were obtained in three independent experiments; CI is the 95% confidence interval.

**Table 3:** Antiviral activity of TDF and mPEG-b-PEPA adducts of TFD.

Compound	SI	SI_add_/SI_TDF_
**TDF**	4836.84	n/a ^1^
**1P3-TFD**	21,771.43	4.5
**1P4-TFD**	65,615.38	13.57
**2P3-TFD**	6590.91	1.36
**2P4-TFD**	13,233.33	2.74

^1^ Not appropriate.

## Data Availability

The data presented in this study are available on request from the corresponding author.

## Supplementary Materials

Supplementary Materials can be found at https://www.mdpi.com/1422-0067/22/1/340/s1. Detailed description of the synthesis of ^t^BuOEP and BHT-Mg catalyst. NMR spectra of ethylene chlorophosphite and ethylene phosphates (Figures S1–S7), polymers **P1**–**P4** (Figures S8–S16) and TFD (Figure S17). Figure S1, ^1^H NMR spectrum (400 MHz, CDCl_3_, 20 °C) of 2-chloro-1,3,2-dioxaphospholane; Figure S2, ^31^P{H} NMR spectrum (162 MHz, CDCl_3_, 20 °C) of 2-chloro-1,3,2-dioxaphospholane; Figure S3, ^1^H NMR spectrum (400 MHz, CDCl_3_, 20 °C) of 2-chloro-2-oxo-1,3,2-dioxaphospholane; Figure S4, ^31^P{^1^H} NMR spectrum (162 MHz, CDCl_3_, 20 °C) of 2-chloro-2-oxo-1,3,2-dioxaphospholane; Figure S5, ^1^H NMR spectrum (400 MHz, CDCl_3_, 20 °C) of *^t^*BuOEP; Figure S6, ^13^C{^1^H} NMR spectrum (101 MHz, CDCl_3_, 20 °C) of *^t^*BuOEP; Figure S7, ^31^P{^1^H} NMR spectrum (162 MHz, CDCl_3_, 20 °C) of *^t^*BuOEP; Figure S8, ^1^H NMR spectrum (400 MHz, CDCl_3_, 20 °C) of **P1**; Figure S9, ^31^P{^1^H} NMR spectrum (162 MHz, CDCl_3_, 20 °C) of **P1**; Figure S10, ^1^H NMR spectrum (400 MHz, CDCl_3_, 20 °C) of **P2**; Figure S11, ^31^P{^1^H} NMR spectrum (162 MHz, CDCl_3_, 20 °C) of **P2**; Figure S12, ^1^H NMR spectrum (400 MHz, D_2_O, 20 °C) of **P3**; Figure S13, ^31^P{^1^H} NMR spectrum (162 MHz, D_2_O, 20 °C) of **P3**; Figure S14, ^1^H NMR spectrum (400 MHz, D_2_O, 20 °C) of **P4**; Figure S15, ^31^P{^1^H} NMR spectrum (162 MHz, D_2_O, 20 °C) of **P4**; Figure S16, ^31^P{^1^H} NMR spectrum (162 MHz, D_2_O, 20 °C) of **P3**/TMP; Figure S17, ^1^H NMR spectrum (400 MHz, CDCl_3_, 20 °C) of TFD; Figure S18, ^1^H (black) and ^31^P (green) NMR spectra (D_2_O, 20 °C) of **1P3-TFD**; Figure S19, ^1^H (black) and ^31^P (green) NMR spectra (D_2_O, 20 °C) of **1P4-TFD**; Figure S20, ^1^H (black) and ^31^P (green) NMR spectra (D_2_O, 20 °C) of **2P3-TFD**; Figure S21, ^1^H (black) and ^31^P (green) NMR spectra (D_2_O, 20 °C) of **2P4-TFD**.
