# Supplementary material for: A New Approach to Developing Long-Acting Injectable Formulations of Anti-HIV Drugs: Poly(Ethylene Phosphoric Acid) Block Copolymers Increase the Efficiency of Tenofovir against HIV-1 in MT-4 Cells
Source: Int J Mol Sci. 2020 Dec 30;22(1):340. doi: 10.3390/ijms22010340 (PMC7795142; doi:10.3390/ijms22010340)
Supplement: Supplementary file 1 [file ijms-22-00340-s001.pdf]

SUPPORTING INFORMATION

S1. Synthesis of cyclic phosphates and BHT-Mg catalyst 2
S2. NMR spectra of polymers 8

S1. Synthesis of cyclic phosphates and BHT-Mg catalyst

2-Chloro-1,3,2-dioxaphospholane

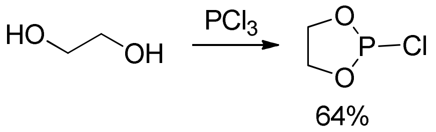

The compound was synthesized via a modified literature protocol [63].

PCl_3 (137.3 g, 1 mol) and CH_2Cl_2 (120 ml) were placed to 500 ml flame-dried three-neck flask, equipped with a dropping funnel and a reflux condenser with a calcium chloride tube. Ethylene glycol (62.1 g, 1 mol) was added dropwise with stirring, argon was bubbled through the solution to remove HCl. After 2 h, the solvent was removed under reduced pressure,and the residue was distilled. The yield was 80.7 g (64%). B. p. 83-84 °C (79-81 Torr), colorless liquid. ^{1}H NMR (400 MHz, $CDCl_3$, 20 °C): δ 4.44 (m, 2H); 4.22 (m, 2H). ^{31}P{H} NMR (162 MHz, $CDCl_3$, 20 °C): δ 167.61. For NMR spectra, see Figures S1, S2.

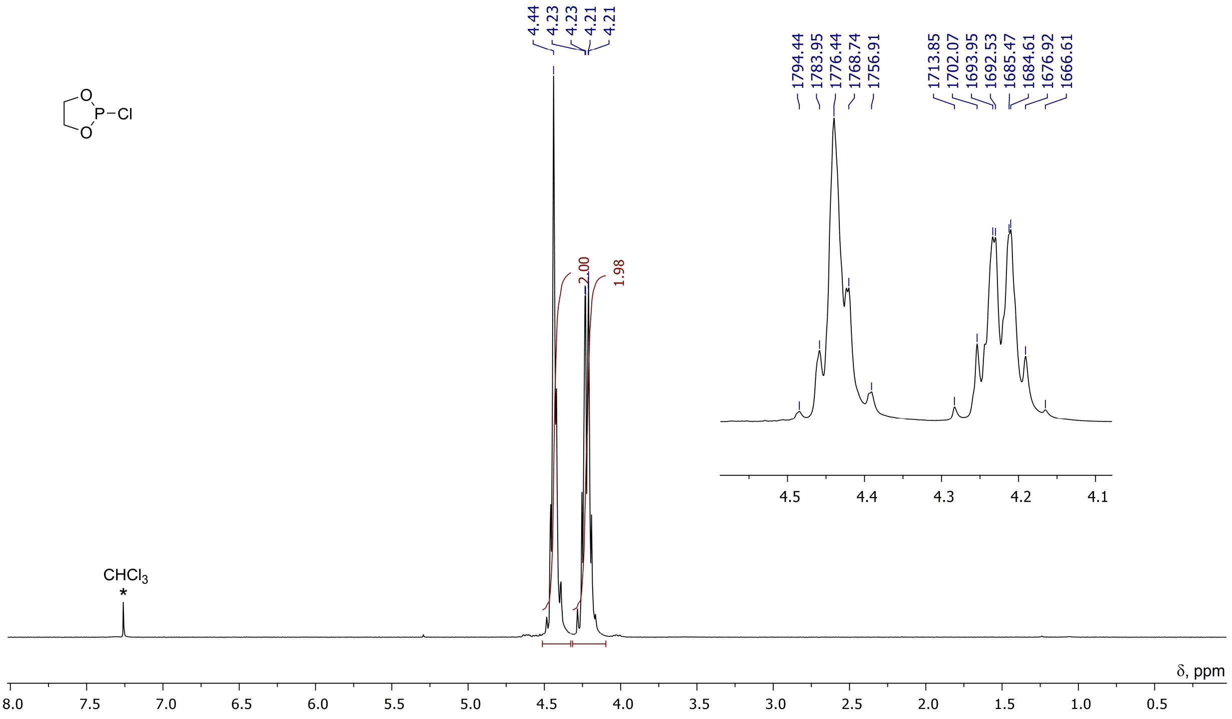

Figure S1. ^{1}H NMR spectrum (400 MHz, $CDCl_3$, 20 °C) of 2-chloro-1,3,2-dioxaphospholane.

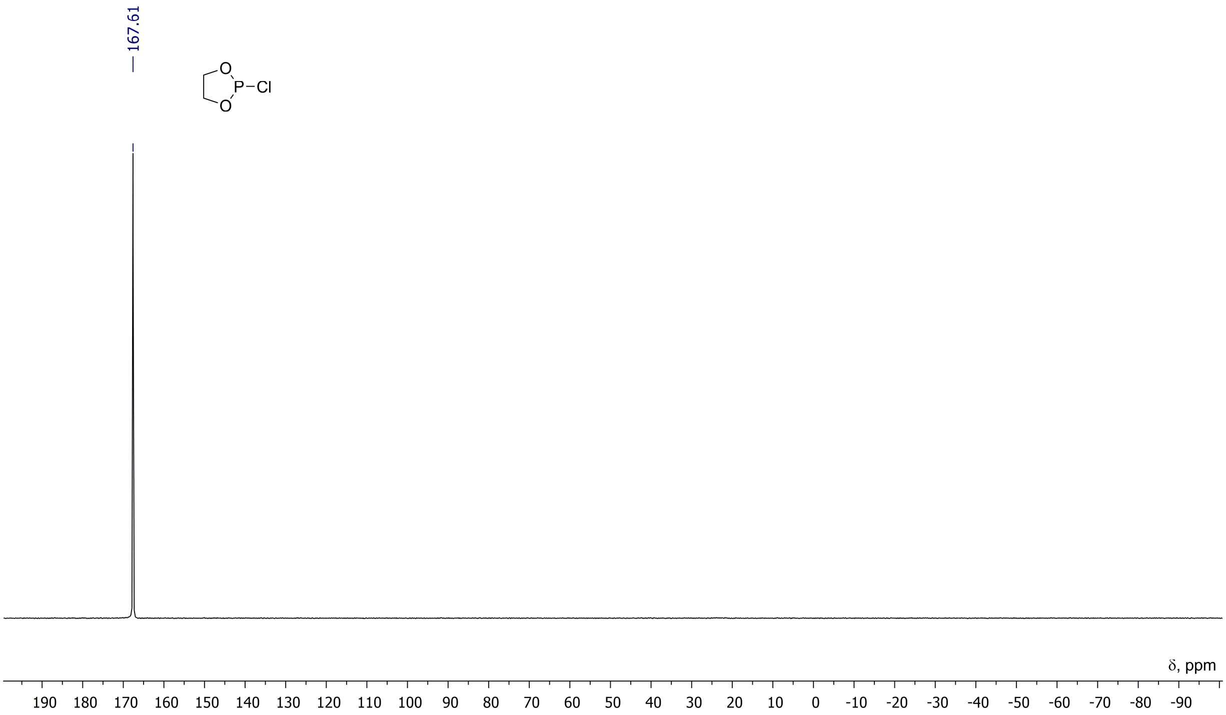

Figure S2. $^{31}P\{H\}$ NMR spectrum (162 MHz, $CDCl_3$, 20 °C) of 2-chloro-1,3,2-dioxaphospholane.

2-Chloro-2-oxo-1,3,2-dioxaphospholane

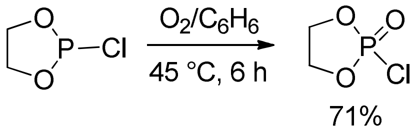

The compound was synthesized according to a modified literature procedure [45]. 2-Chloro-1,3,2-dioxaphospholane (50 g, 0.4 mol) and dry benzene (200 ml) were placed to flame-dried 500 ml three-neck flask, equipped with a reflux condenser, and the mixture was heated to 50 °C. A stream of oxygen was passed through the solution for 12 h with stirring. The solvent was removed under reduced pressure, and the residue was purified by distillation *in vacuo*. The yield was 40.1 g (71%). B. p. 79-80 °C (0.4 Torr), colorless liquid.

^{1}H NMR (400 MHz, $CDCl_3$, 20 °C): δ 4.61-4.48 (m, 4H).

$^{31}P\{H\}$ NMR (162 MHz, $CDCl_3$, 20 °C): δ 22.81.

For NMR spectra, see Figures S3, S4.

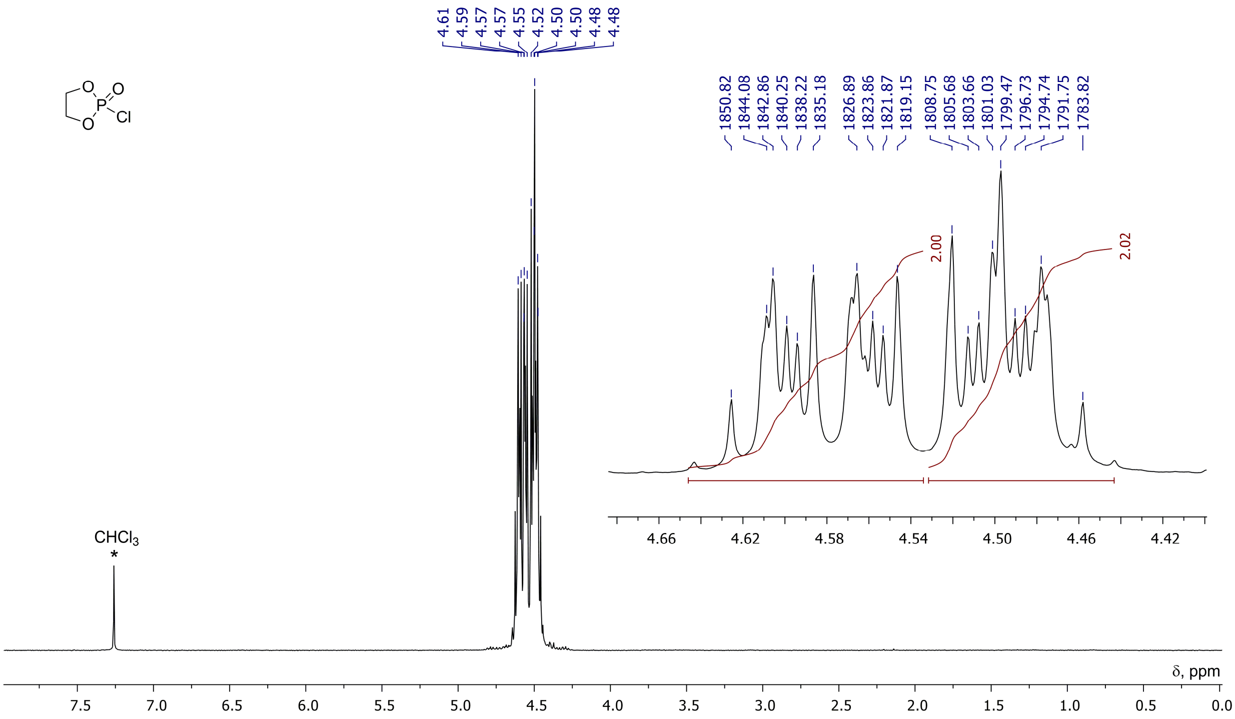

Figure S3. ^{1}H NMR spectrum (400 MHz, $CDCl_3$, 20 °C) of 2-chloro-2-oxo-1,3,2-dioxaphospholane.

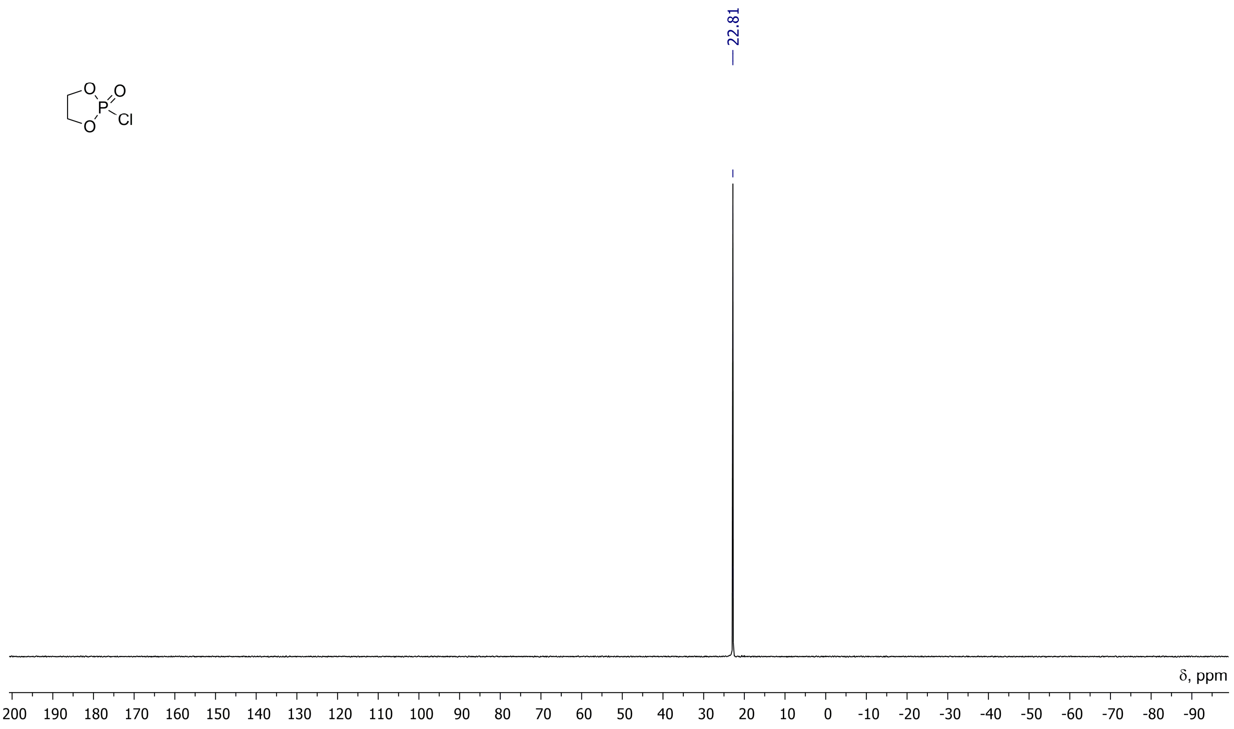

Figure S4. $^{31}P\{^{1}H\}$ NMR spectrum (162 MHz, $CDCl_3$, 20 °C) of 2-chloro-2-oxo-1,3,2-dioxaphospholane.

2-*tert*-Butoxy-2-oxo-1,3,2-dioxaphospholane (*tert*-butyl ethylene phosphate, ^{t}BuOEP)

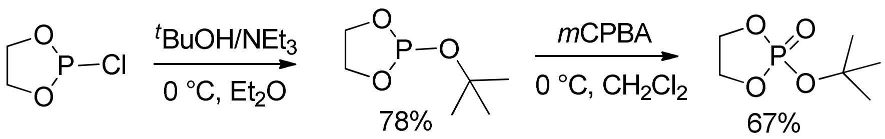

The compound was synthesized via a modified literature protocol [17].

2-tert-Butoxy-1,3,2-dioxaphospholane.

Dry *tert*-butanol (38.6 g, 0.52 mol), triethylamine (78 ml, 0.52 mol) and diethyl ether (500 ml) were placed into flame-dried 1 l three-neck flask, equipped with a dropping funnel. A solution of 2-chloro-oxo-1,3,2-dioxaphospholane (66.0 g, 0.52 mol) in dry ether (60 ml) was added dropwise under stirring at 0 °C. The mixture was allowed to warm to room temperature and stirred overnight. Triethylammonium chloride was filtered off, and the filtrate was concentrated *in vacuo*. The residue was purified by distillation under reduced pressure. The yield was 67.1 g (78%). B. p. 70-75°C (13 Torr), colorless liquid. ^{1}H NMR (400 MHz, CDCl$_3$, 20 °C): δ 4.14 (m, 2H, OC$\underline{H}_2$); 3.88 (m, 2H, OC$\underline{H}_2$); 1.47 (s, 9H, O-C(C$\underline{H}_3$)$_3$. ^{31}P{H} NMR (162 MHz, CDCl$_3$, 20 °C): δ 134.57 (s).

2-tert-Butoxy-2-oxo-1,3,2-dioxaphospholane.

2-*tert*-Butoxy-1,3,2-dioxaphospholane (16.4 g, 0.1 mol) in dry dichloromethane (200 ml) were placed into flame-dried 500 ml three-neck flask, equipped with a dropping funnel. A solution of *m*-chloroperbenzoic acid (~0.11 mol) prepared by drying of the mixture of commercial 70-77% *m*-chloroperbenzoic acid (26 g) in 200 ml of dichloromethane over MgSO$_4$, was added dropwise under stirring at 0 °C within 4 h. The resulting precipitate of *m*-chlorobenzoic acid was removed by filtration. The filtrate was treated with the aq. solutions of 15% K$_2$CO$_3$ (2×80 ml) and 20% Na$_2$S$_2$O$_3$ aqueous solution (3×40 ml), dried over MgSO$_4$ and concentrated *in vacuo* to give white crystalline substance (M. p. 28 °C). The yield was 12.2 g (67%). ^{1}H NMR (400 MHz, CDCl$_3$, 20 °C): δ 4.33 (m, 2H, OC$\underline{H}_2$); 4.26 (m, 2H, OC$\underline{H}_2$); 1.47 (s, 9H, O-C(C$\underline{H}_3$)$_3$. ^{13}C{H} NMR (101 MHz, CDCl$_3$, 20 °C): δ 84.08 (d, $^{2}J_{CP}$ = 7.2 Hz, 1C, O-$\underline{C}$(CH$_3$)$_3$); 65.55 (d, $^{2}J_{CP}$ = 2.3 Hz, 2C, O$\underline{C}$H$_2$CH$_2$O); 29.69 (d, $^{3}J_{CP}$ = 4.4 Hz, 3C, O-C($\underline{C}$H$_3$)$_3$). ^{31}P{H} NMR (162 MHz, CDCl$_3$, 20 °C): δ 13.21 (s).

For NMR spectra, see Figures S5–S7.

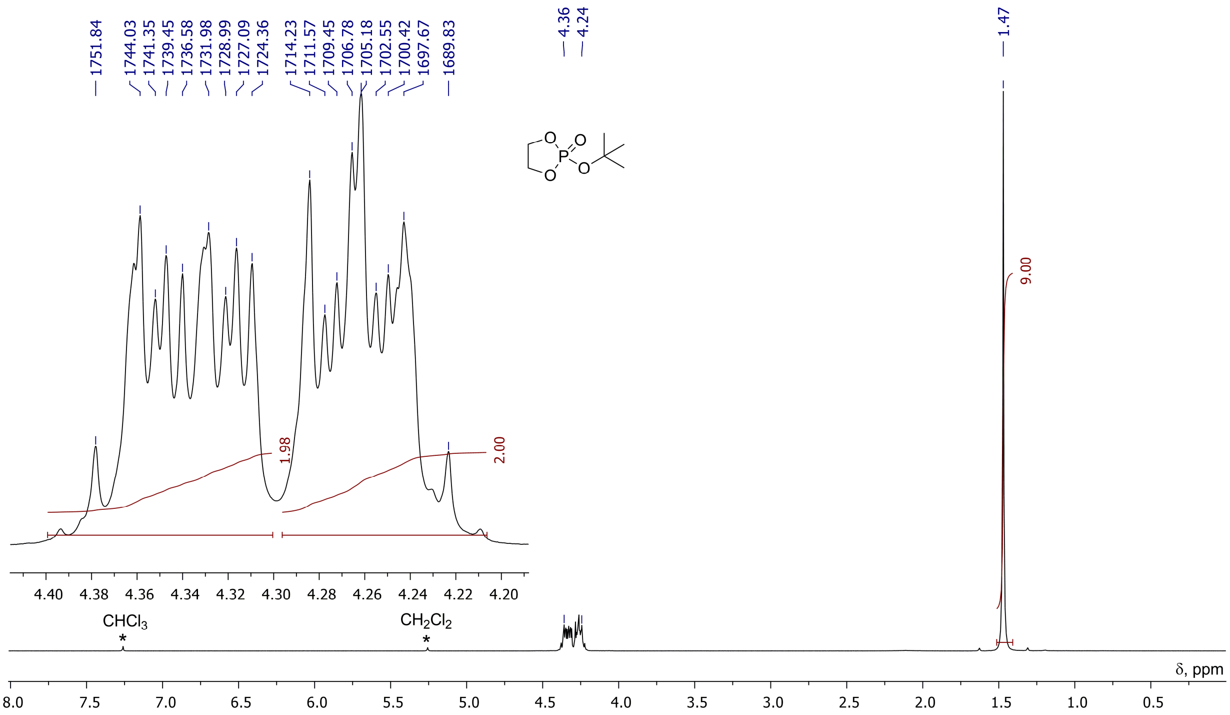

Figure S5. ^{1}H NMR spectrum (400 MHz, $CDCl_3$, 20 °C) of ^{t}BuOEP.

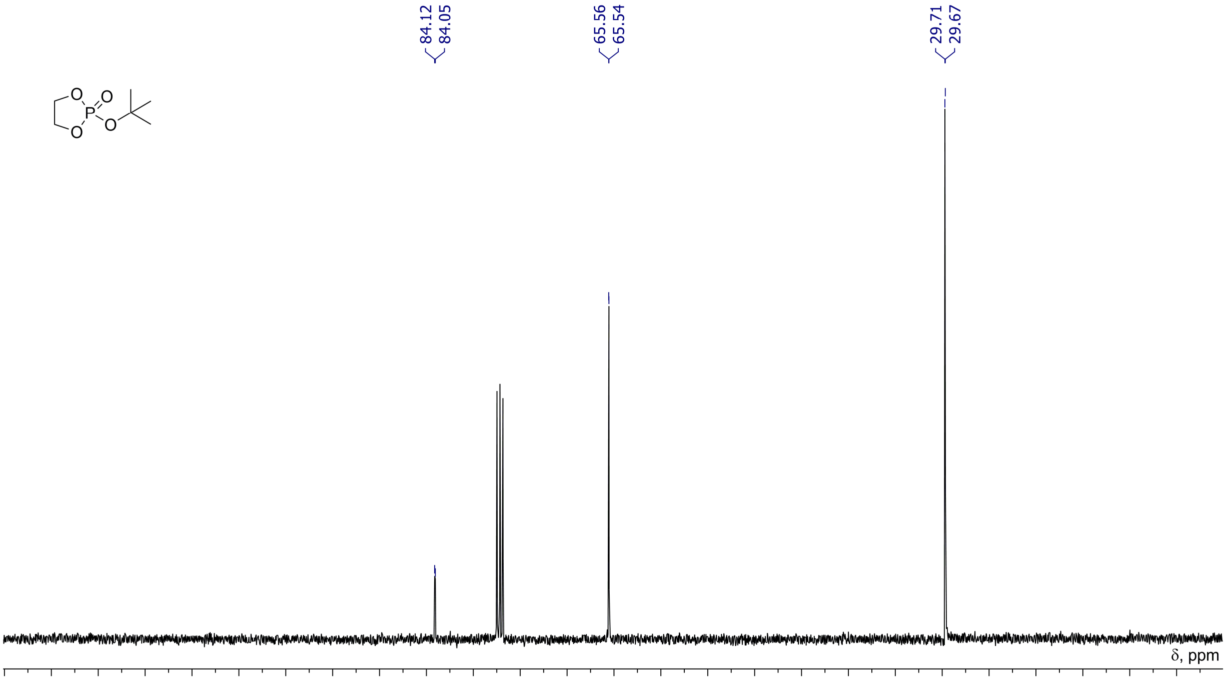

Figure S6. $^{13}C\{^{1}H\}$ NMR spectrum (101 MHz, $CDCl_3$, 20 °C) of ^{t}BuOEP.

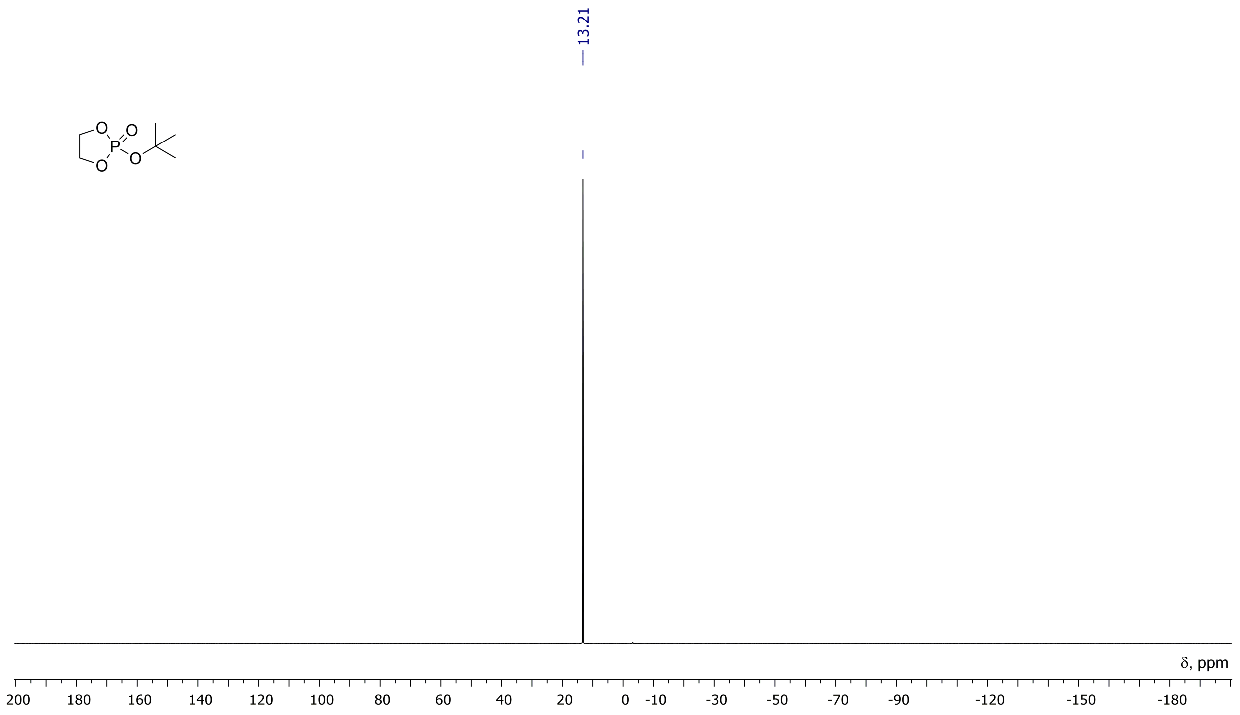

Figure S7. $^{31}P\{^{1}H\}$ NMR spectrum (162 MHz, $CDCl_3$, 20 °C) of ^{t}BuOEP.

Note that high grade of purity of ^{t}BuOEP is essential for further polymerization experiments.

[(BHT)Mg(μ-BnO)(THF)]$_2$

The compound was synthesized via a modified literature protocol [64].

BHT-H (0.22 g, 1 mmol) was placed into an ampoule filled with argon. Heptane (1 ml) was injected with a syringe through a septum. Upon BHT-H dissolution, the ampoule was cooled to -10 °C. Dibutylmagnesium (1 ml of 1 M in heptane, 1 mmol) was transferred into the ampoule while stirring the solution. After warming to room temperature and stirring for 4 h, the as-formed crystals were washed with 2 ml of heptane and dried under vacuum. The obtained solid was dissolved in THF (2 ml). The resulting solution was evaporated under reduced pressure to a residual volume of 0.5 ml. The as-formed crystalline precipitate was filtered, washed with 1 ml of a 1:1 (v/v) THF/*n*-heptane mixture, and dried *in vacuo*. The yield was 0.38 g (86 %). ^{1}H NMR (400 MHz, THF-d_8, 20 °C): δ 6.72 (s, 2H, CH(Ar)), 2.09 (s, 3H, Me(Ar)), 1.51 (qv, 2H, $CH_3CH_2C\underline{H_2}$), 1.36 (s, 18H, ^{t}Bu), 1.24 (sex, 2H, CH_3CH_2), 0.84 (t, 3H, $C\underline{H_3}CH_2$), 0.49 (m, 2H, CH_2Mg). ^{13}C-$\{^{1}H\}$ NMR (100 MHz, THF-d_8, 20 °C): δ 161.5 (C(Ar)-O), 137.0 (Ar), 125.0 (Ar), 119.2 (Ar), 35.1 ($(CH_3)_3\underline{C}$), 33.5 (CH_2CH_2), 32.5 (CH_2CH_2), 30.7 ($(\underline{C}H_3)_3C$), 21.3 (Me(Ar)), 14.1 (Me(Bu)), 8.2 (CH_2Mg).

S2. NMR spectra of polymers

NMR spectra demonstrate the given ratio of mPEG and ethylene phosphate fragments confirming the living character of the formation of mPEG-*b*-polyphosphate comolymers.

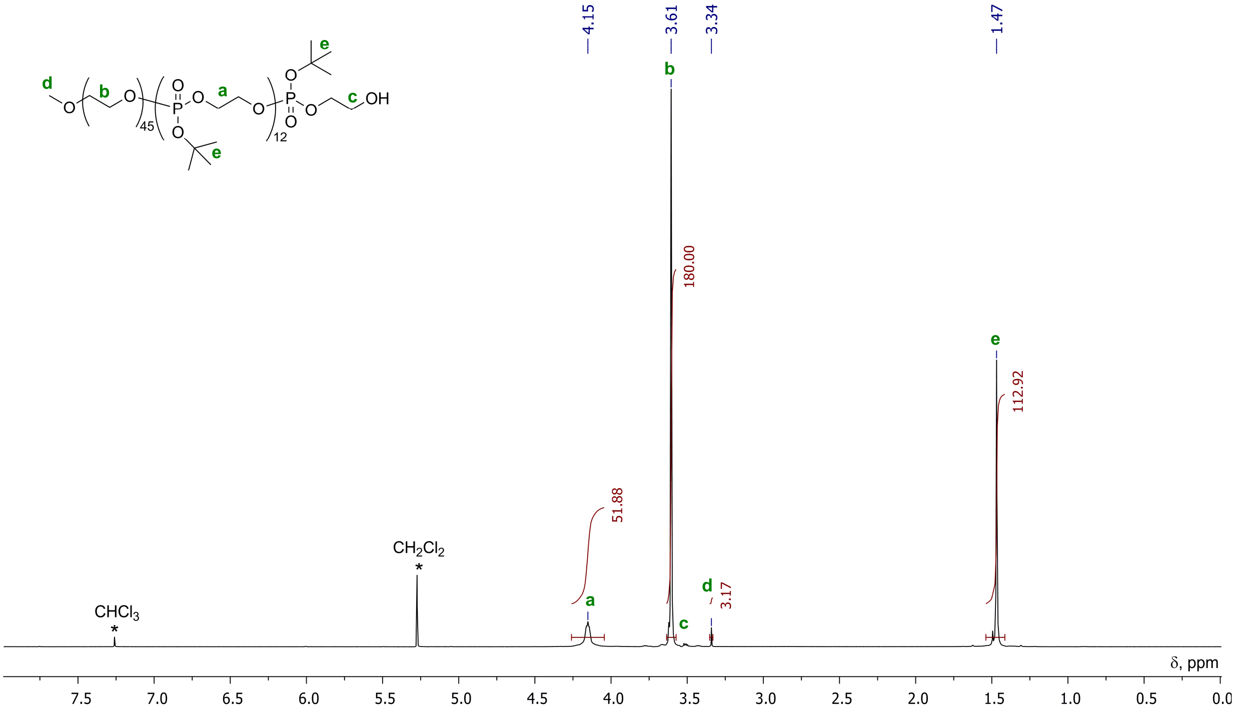

Figure S8. ^{1}H NMR spectrum (400 MHz, $CDCl_3$, 20 °C) of **P1**.

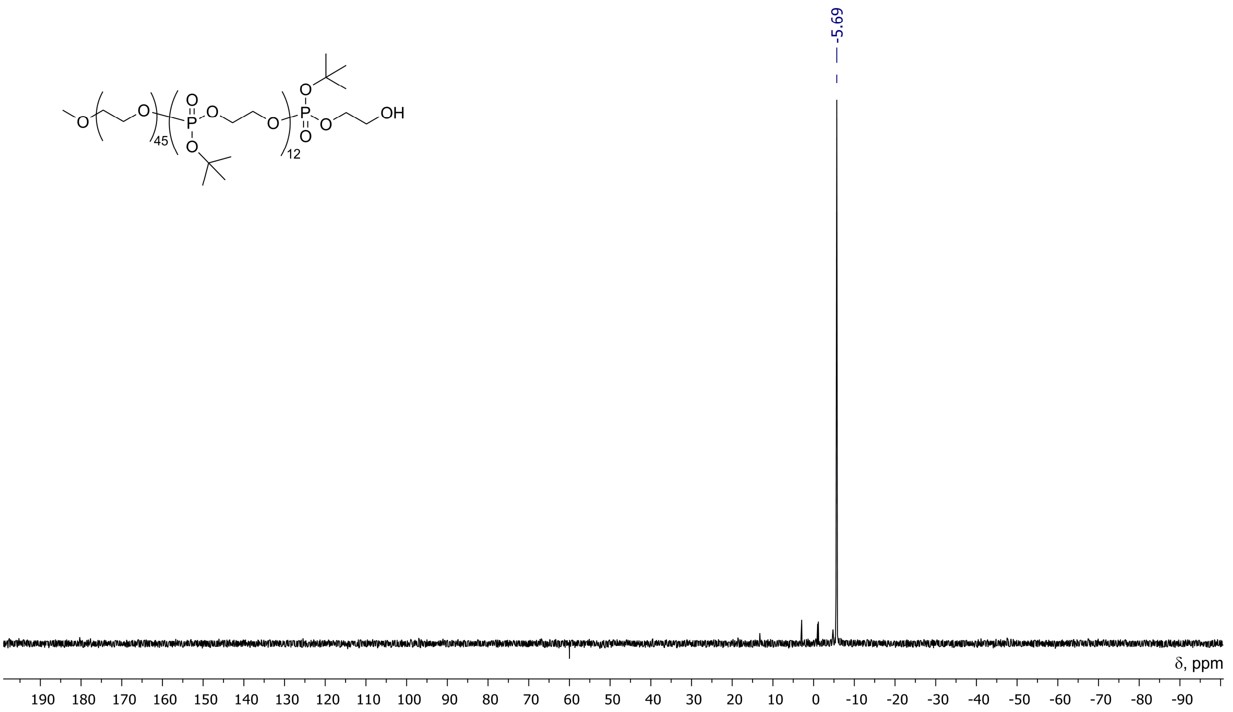

Figure S9. $^{31}P\{^{1}H\}$ NMR spectrum (162 MHz, $CDCl_3$, 20 °C) of **P1**.

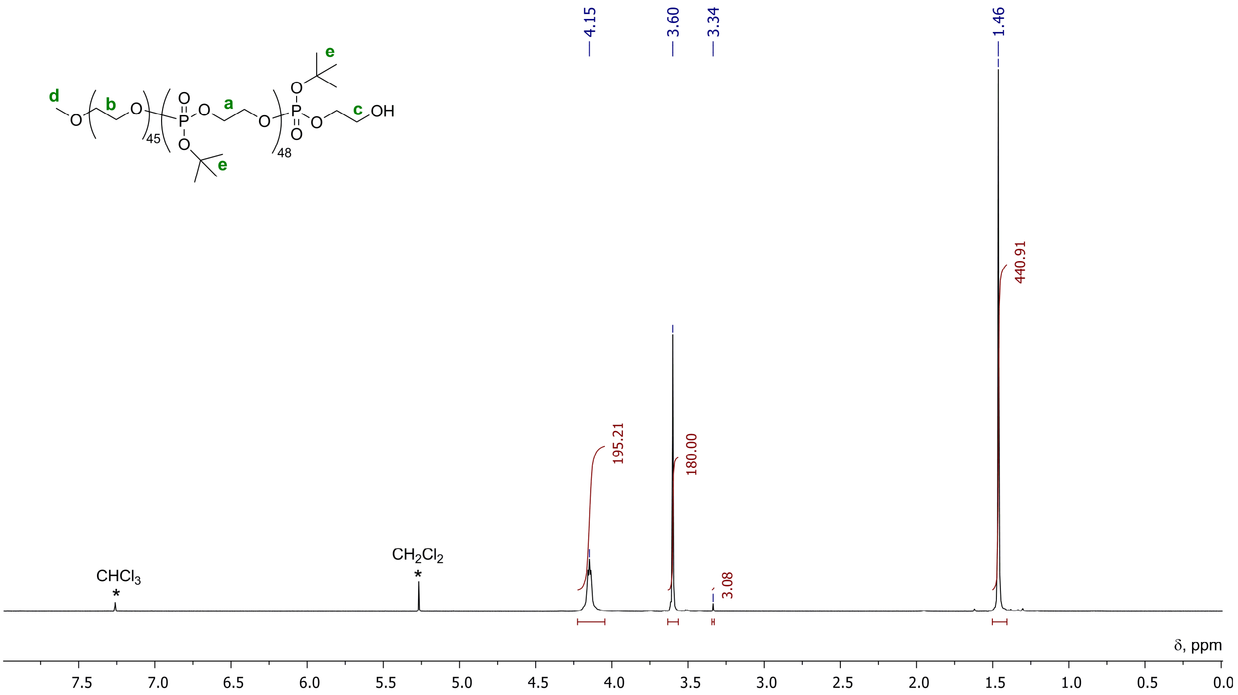

Figure S10. ^{1}H NMR spectrum (400 MHz, $CDCl_3$, 20 °C) of **P2**.

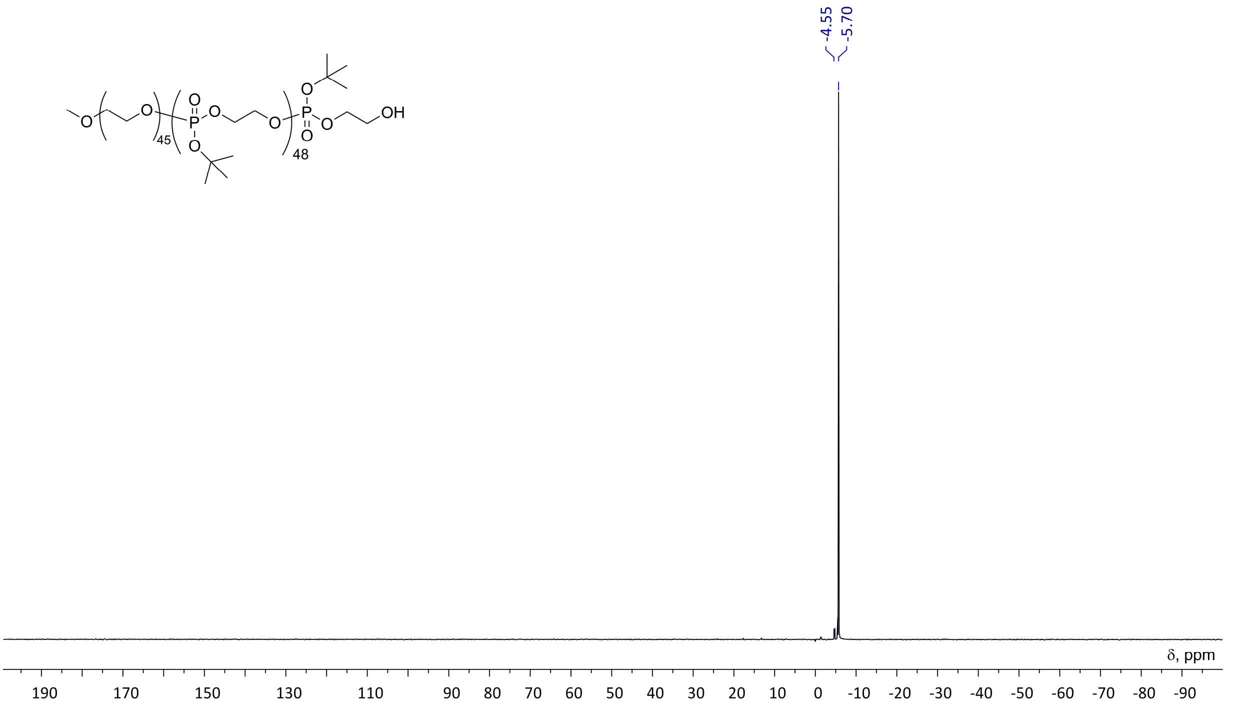

Figure S11. $^{31}P\{^{1}H\}$ NMR spectrum (162 MHz, $CDCl_3$, 20 °C) of **P2**.

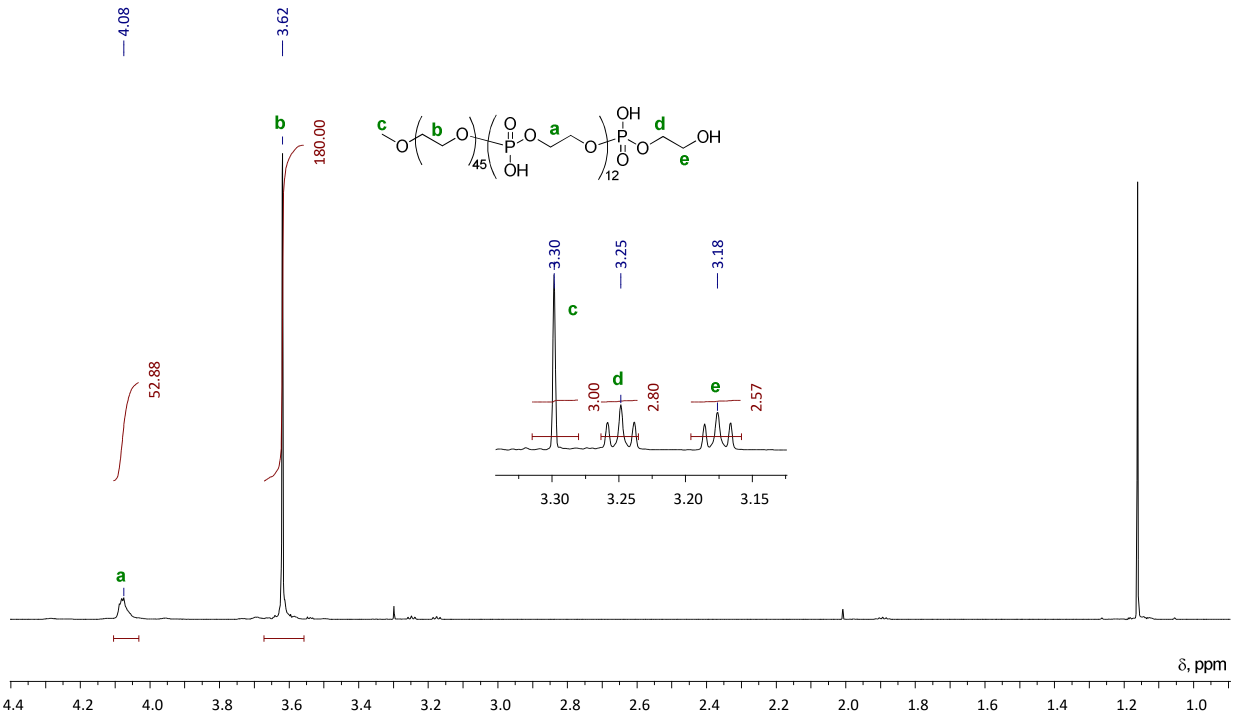

Figure S12. ^{1}H NMR spectrum (400 MHz, D_2O, 20 °C) of **P3**.

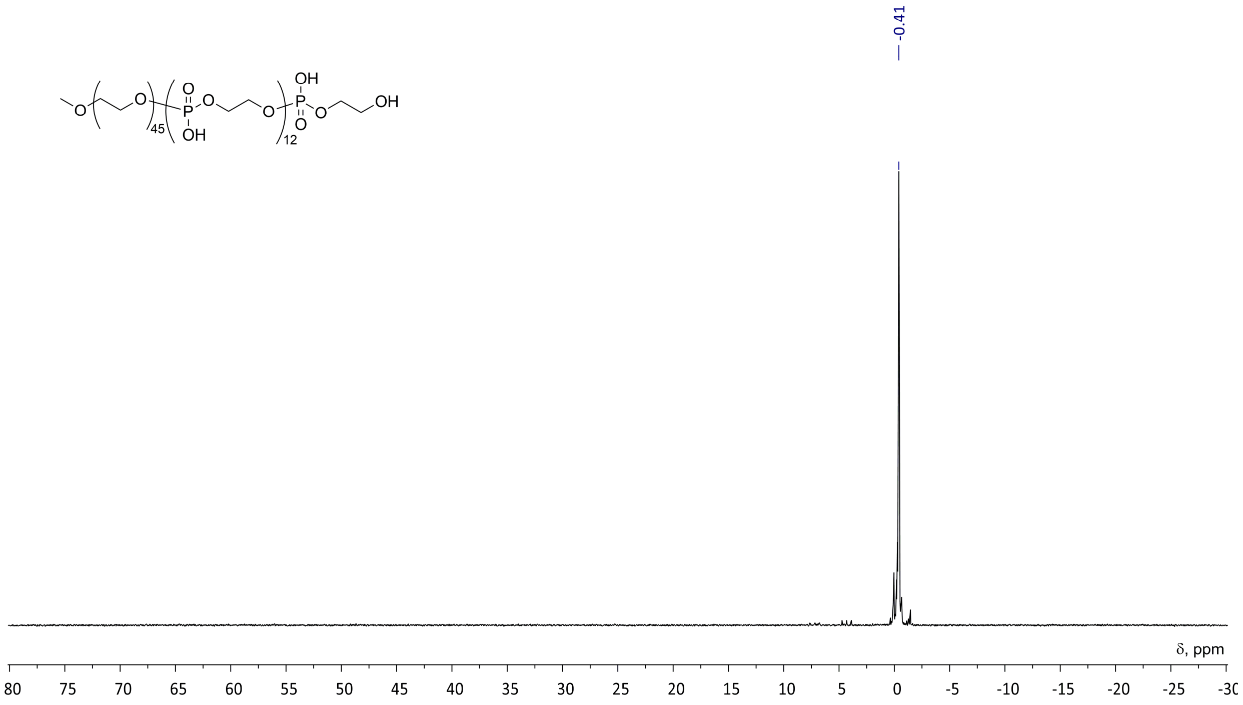

Figure S13. $^{31}P\{^{1}H\}$ NMR spectrum (162 MHz, D_2O, 20 °C) of **P3**.

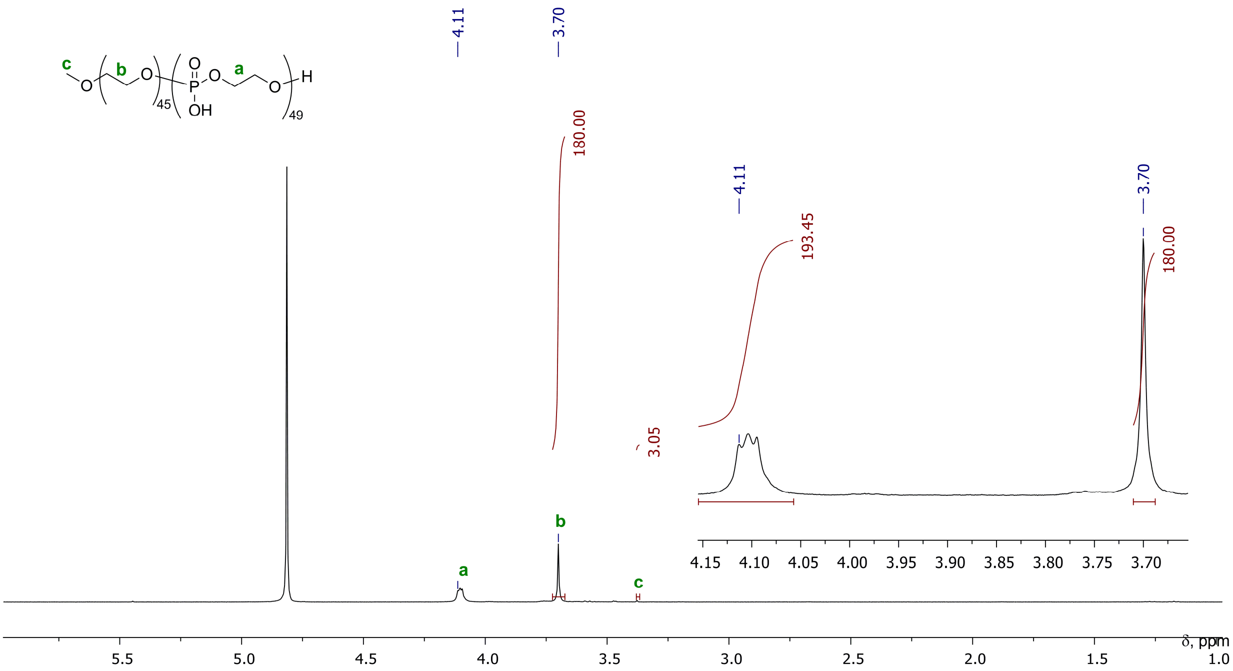

Figure S14. ^{1}H NMR spectrum (400 MHz, D_2O, 20 °C) of **P4**.

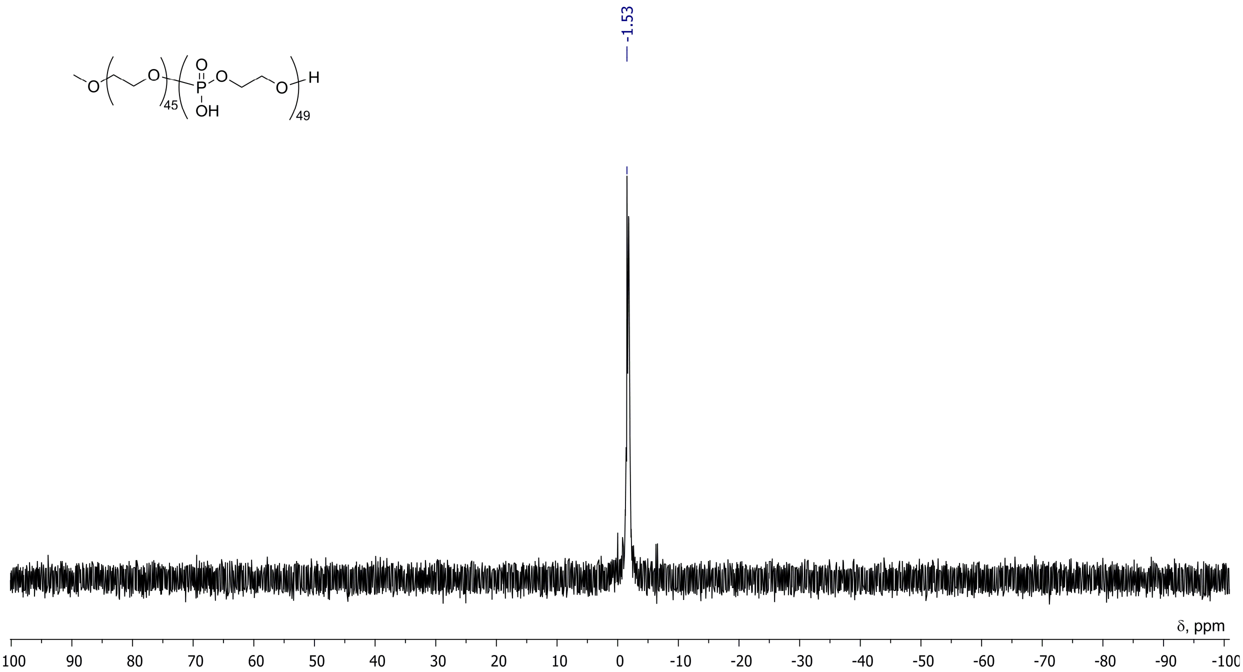

Figure S15. $^{31}P\{^{1}H\}$ NMR spectrum (162 MHz, D_2O, 20 °C) of **P4**.

^{31}P NMR spectroscopy was used for the determination of the concentration of phosphate groups in aqueous mPEG-*b*-PEPA solutions. Calculated amount of TMP (corresponding to [TMP]/[–$OCH_2CH_2OP(O)O$] ratio of 10:1) was added to the solution, and ^{31}P NMR spectra was registered. This method was also used for **P4** and for TFD adducts **1P3-TFD**, **1P4-TFD**, **2P3-TFD**, **2P4-TFD** (Figures S18–S21).

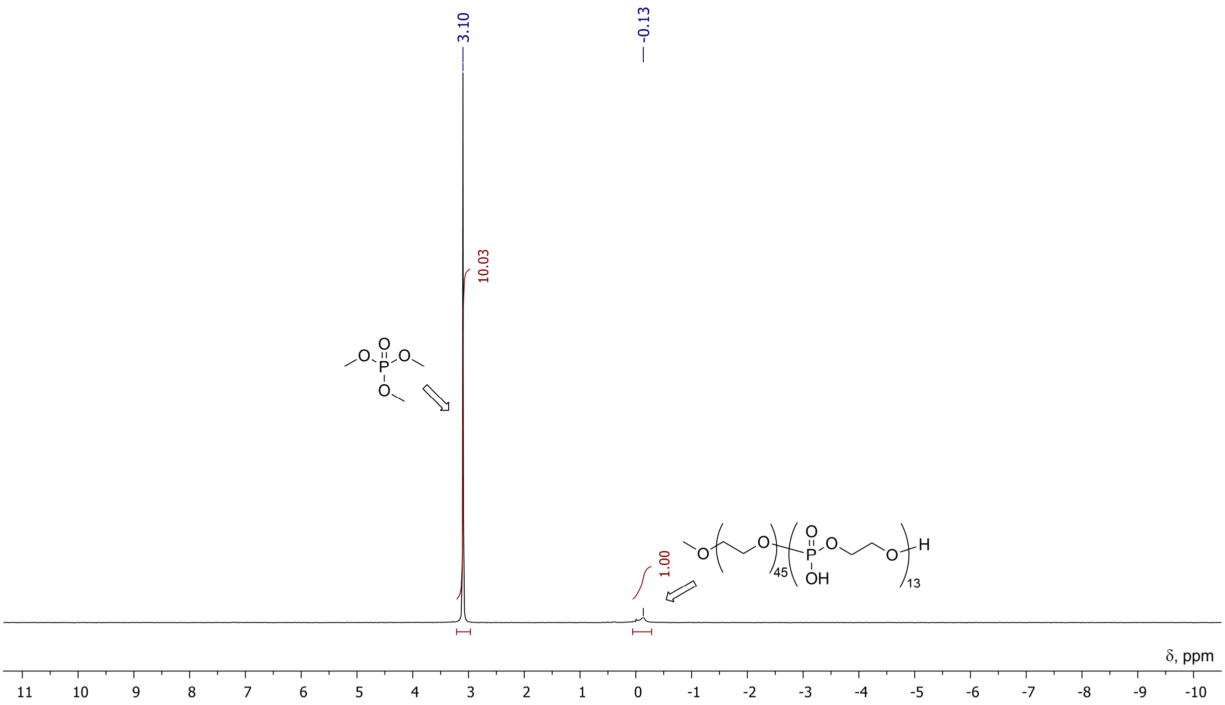

Figure S16. $^{31}P\{^1H\}$ NMR spectrum (162 MHz, D_2O, 20 °C) of **P3**/TMP.

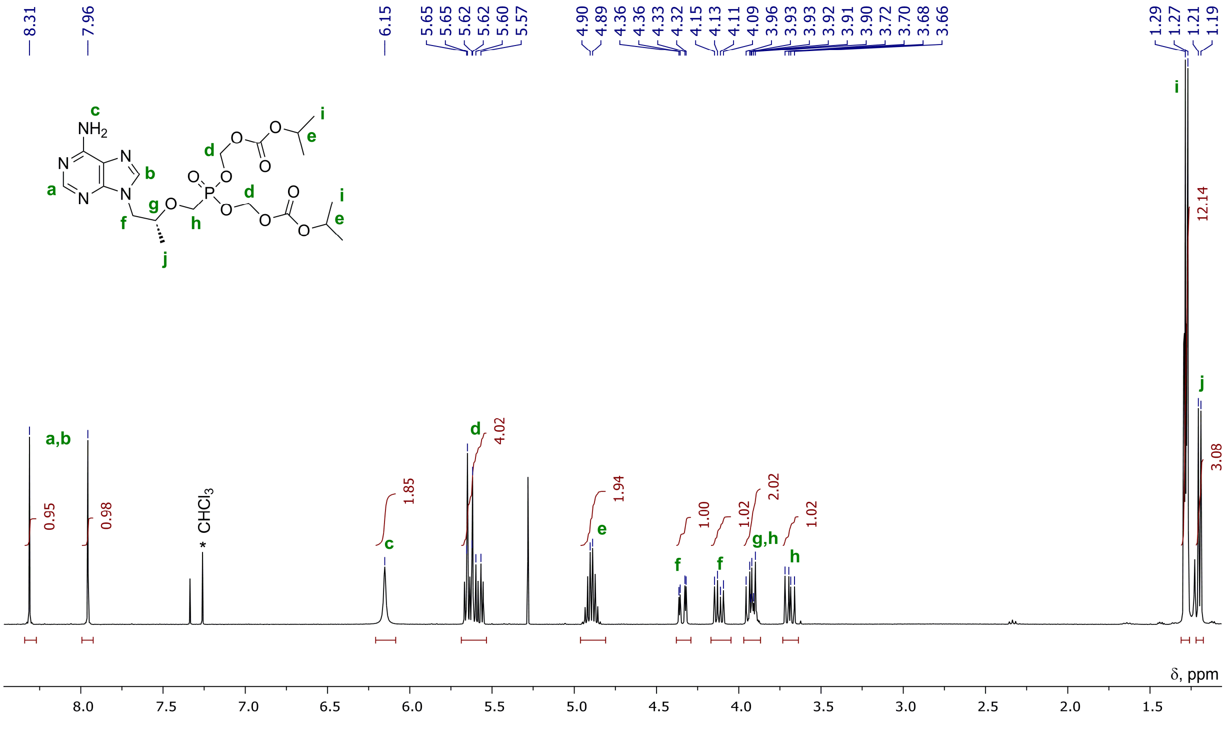

Figure S17. ^1H NMR spectrum (400 MHz, $CDCl_3$, 20 °C) of TFD.

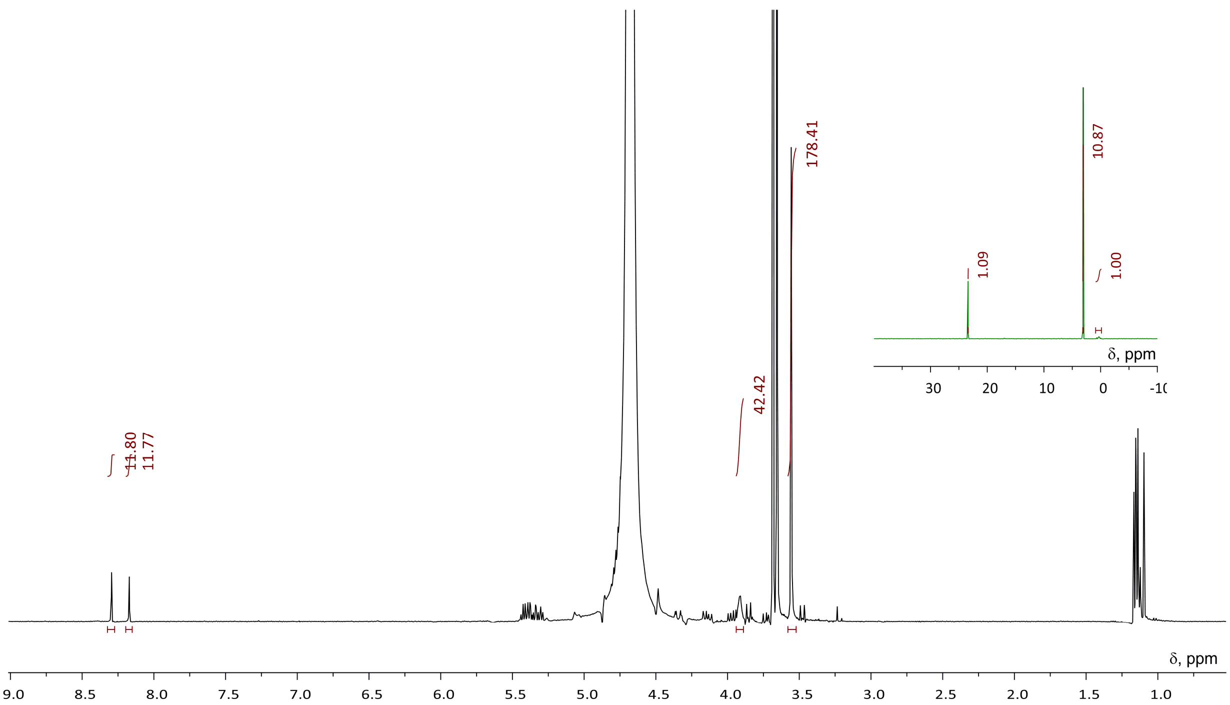

Figure S18. ^{1}H (black) and ^{31}P (green) NMR spectra (D_2O, 20 °C) of **1P3-TFD**.

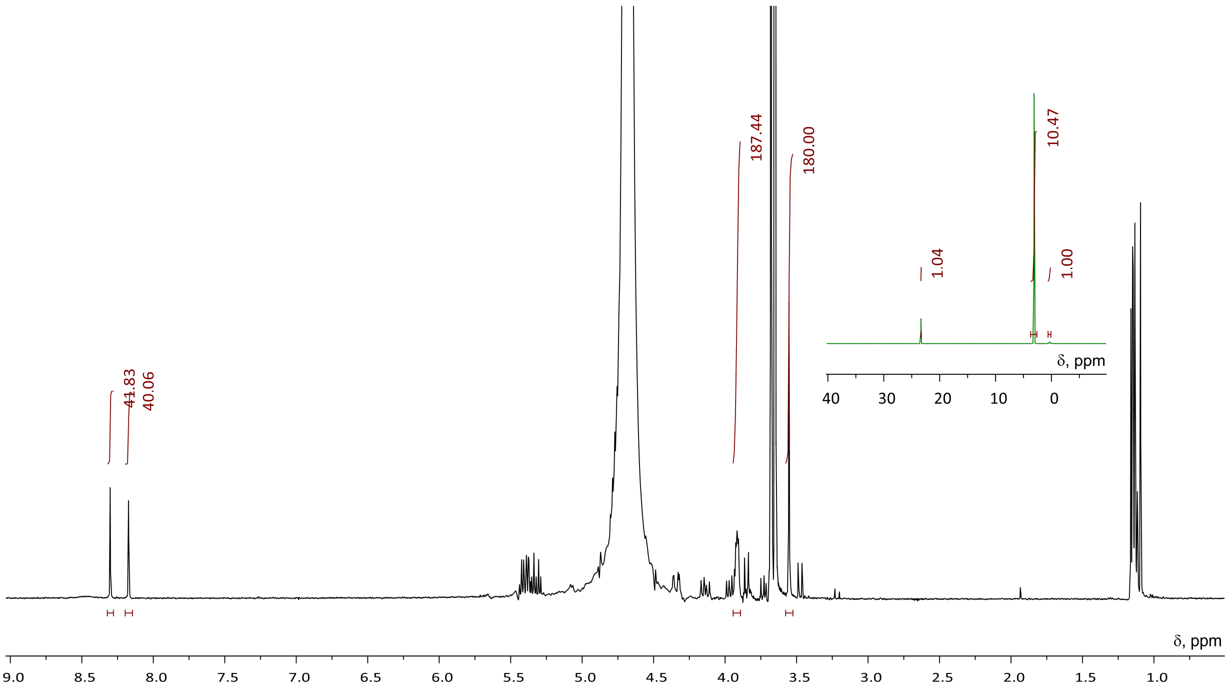

Figure S19. ^{1}H (black) and ^{31}P (green) NMR spectra (D_2O, 20 °C) of **1P4-TFD**.

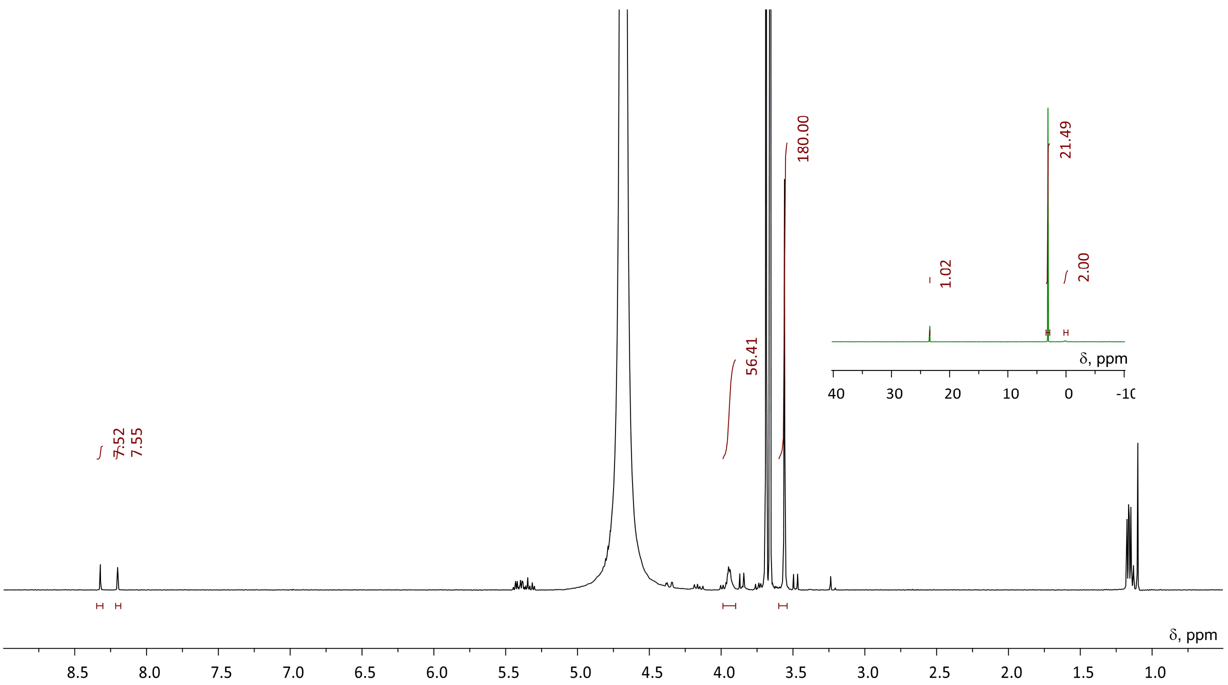

Figure S20. ^{1}H (black) and ^{31}P (green) NMR spectra (D_2O, 20 °C) of **2P3-TFD**.

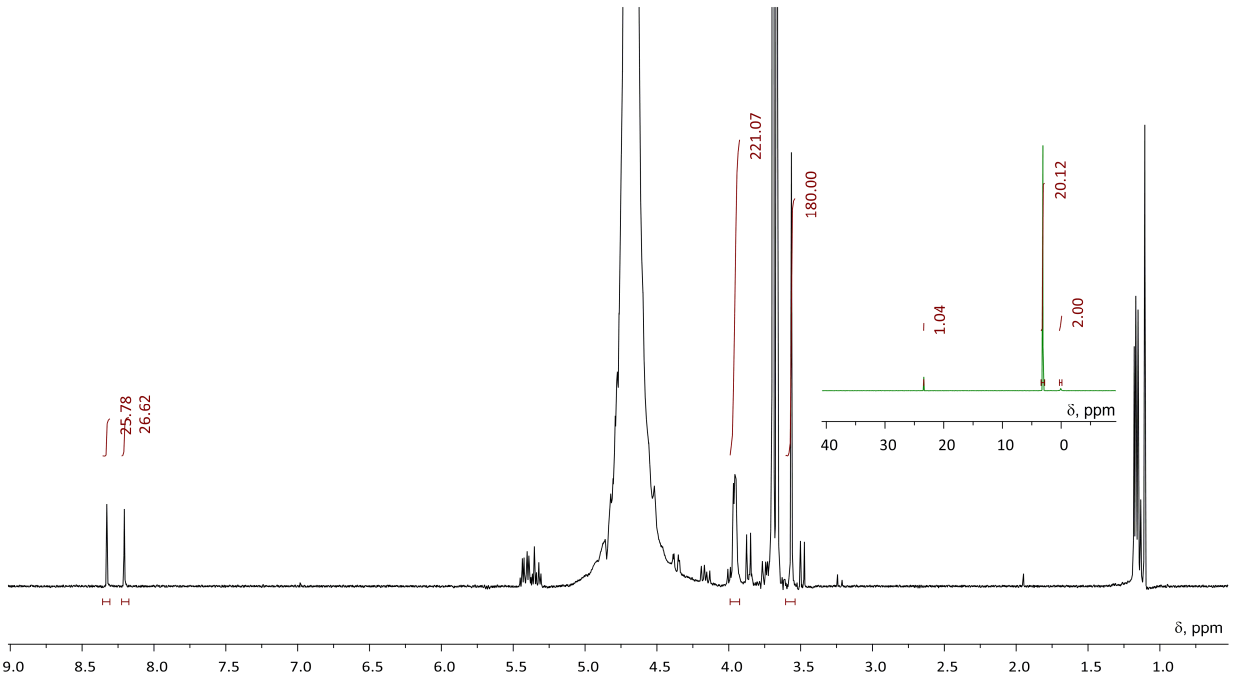

Figure S21. ^{1}H (black) and ^{31}P (green) NMR spectra (D_2O, 20 °C) of **2P4-TFD**.
